# Greenotyper: Image-Based Plant Phenotyping Using Distributed Computing and Deep Learning

**DOI:** 10.3389/fpls.2020.01181

**Published:** 2020-08-07

**Authors:** Marni Tausen, Marc Clausen, Sara Moeskjær, ASM Shihavuddin, Anders Bjorholm Dahl, Luc Janss, Stig Uggerhøj Andersen

**Affiliations:** ^1^ Bioinformatics Research Centre, Aarhus University, Aarhus, Denmark; ^2^ Department of Molecular Biology and Genetics, Aarhus University, Aarhus, Denmark; ^3^ Image Analysis & Computer Graphics, DTU Compute, Lyngby, Denmark; ^4^ EEE Department, Green University of Bangladesh (GUB), Dhaka, Bangladesh

**Keywords:** deep learning, plant phenotyping, image detection, Raspberry Pi, greenness measures, object detection, segmentation, software

## Abstract

Image-based phenotype data with high temporal resolution offers advantages over end-point measurements in plant quantitative genetics experiments, because growth dynamics can be assessed and analysed for genotype-phenotype association. Recently, network-based camera systems have been deployed as customizable, low-cost phenotyping solutions. Here, we implemented a large, automated image-capture system based on distributed computing using 180 networked Raspberry Pi units that could simultaneously monitor 1,800 white clover (*Trifolium repens*) plants. The camera system proved stable with an average uptime of 96% across all 180 cameras. For analysis of the captured images, we developed the Greenotyper image analysis pipeline. It detected the location of the plants with a bounding box accuracy of 97.98%, and the U-net-based plant segmentation had an intersection over union accuracy of 0.84 and a pixel accuracy of 0.95. We used Greenotyper to analyze a total of 355,027 images, which required 24–36 h. Automated phenotyping using a large number of static cameras and plants thus proved a cost-effective alternative to systems relying on conveyor belts or mobile cameras.

## Introduction

Understanding plant genetic effects driving phenotypic differences requires extensive amounts of phenotypic data. Traditional phenotyping approaches are often limited by the time required for data collection and can suffer from batch effects if multiple people are involved in phenotype assessment. In contrast, automated phenotyping systems can potentially generate large amounts of unbiased phenotype measurements in a cost-effective manner.

Quantifying yield and growth rate is instrumental for identifying productive genotypes in plant breeding (Wang et al., 2004; Lee and Tollenaar, 2007; Rahman et al., 2009; Fischer and Edmeades, 2010; Rivano et al., 2013). Plant yield can relatively easily be quantified by registering biomass or grain weight post-harvest (Serfaty et al., 2013). Assessing growth rate is more difficult as it requires multiple measurements during plant cultivation (Walter et al., 2015), and manual daily measurements may be impractical in large-scale studies.

Complex plant phenotyping systems that rely on advanced robotics can help to address this issue by providing large amounts of high-quality image data from advanced cameras. Many of these systems use conveyor belts to move plants to a high-quality camera (Tisné et al., 2013; Fujita et al., 2018), whereas others rely on a mobile camera (Lee et al., 2018). However, parts for such systems are expensive, their maintenance and construction is challenging, and they often require expert assembly and manufacturing.

Recently, single board embedded computers with camera modules, such as the Raspberry Pi (RPi), have been used as customizable, scalable, and inexpensive solutions for image capture in plant phenotyping (Minervini et al., 2017; Tovar et al., 2018; Grindstaff et al., 2019). Phenotyping systems with networked RPis are modular in nature, and their operation can be automated with simple software solutions, allowing continuous collection of large amounts of phenotypic data. RPis have also been coupled with environmental monitoring components to combine image capture with measurements of temperature, humidity, and light intensity (Grindstaff et al., 2019).

Once images have been captured, the next challenge is to extract relevant plant features, such as the projected plant area, through image segmentation. In large-scale experiments, multiple plants will often be monitored by one camera, necessitating detection and segmentation of multiple plants from each image. This is accomplished by a number of image analysis pipelines developed for quantification of *Arabidopsis thaliana* rosette growth (De Vylder et al., 2012; Gehan et al., 2017; Minervini et al., 2017), but these are not necessarily easily applied to other plant species. Methods also exist for segmentation of complex canopies, but these are not capable of handling multi-plant images (Guo et al., 2017; Zhang and Xu, 2018).

Machine learning-based approaches provide interesting alternative approaches to image segmentation. Supervised segmentation approaches such as EasyPCC (Guo et al., 2017) and ilastik (Sommer et al., 2011) require supervision to separate the object of interest from the background. Supervised methods perform

well but require a comprehensive set of training data to match the variation expected in real-life data. An unsupervised plant segmentation method that uses k-means clustering with an EM (expectation and maximization) algorithm has also been reported (Al-Shakarji et al., 2017). In addition, deep learning general object detection approaches, such as R-CNN networks (Huang et al., 2017), have been used for disease quantification (Fuentes et al., 2017) and for detection of maize plants in field trials using Lidar-imaging (Jin et al., 2018).

Following successful segmentation, a wide range of features can be extracted from the individual plant masks. For instance, plant color has previously been used to estimate nitrogen deficiency in legumes (Wiwart et al., 2009). Different approaches using indices based on RGB (Tewari et al., 2013) or estimating euclidean distances using HSL and CIELAB color spaces have been applied (Wiwart et al., 2009), and the mean of the hue component from the HSV/HSL/IHLS color spaces has previously been found to correlate strongly with nitrogen content in tomato seedlings (Mata-Donjuan et al., 2012).

Here, we present our solution for building and managing a large-scale RPi camera system and the development of the accompanying deep learning–based Greenotyper image analysis pipeline. We used the system for continuous monitoring of the projected area and hue of 1800 white clover plants in a greenhouse setting.

## Methods

The methods section is divided into three sections; the camera system, the image analysis pipeline, and the experimental setup. The camera system refers to the physical camera setup in the greenhouse and the management of this system. The image analysis pipeline includes the processing of the produced images and production of the final results. The experimental setup describes the experiments performed to test the camera system and the image analysis system, and the experimental setup the system was used to monitor.

### Camera System

#### Introduction

**Figure 1:**
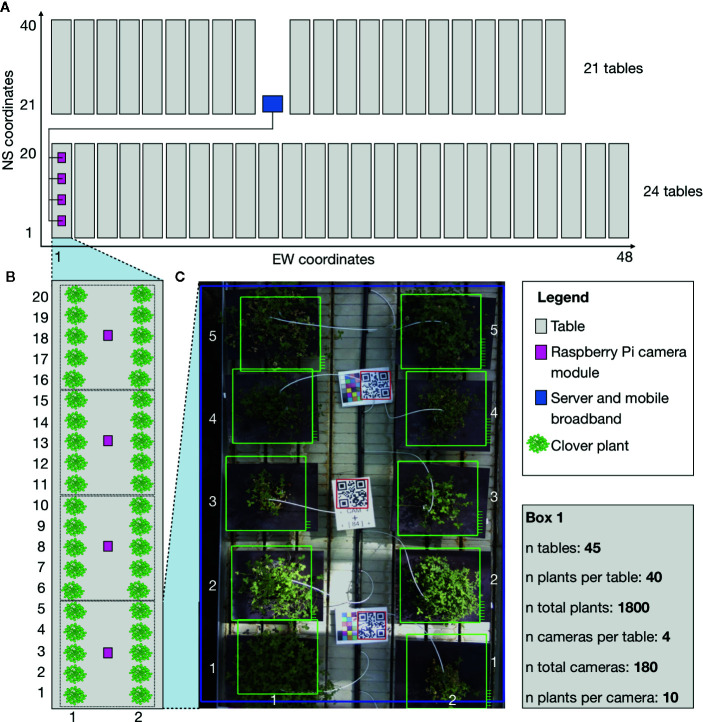
Schematic showing the general setup of the phenotyping system and experimental setup. The schematic corresponds to the physical setup in the greenhouse. The grey boxes are moveable tables. The pink boxes are RPi camera modules fixed (2 m) over the tables in a fixed position. The blue box indicates the location of the central computer, which all of the camera modules were connected to. Green indicates locations of clover plants that were studied in the experiment. The camera modules are only shown for a single table, but camera modules were mounted over all tables with four per table. **(A)** A full schematic layout of the greenhouse with relative table placements. EW (East-West) coordinates and NS (North-South) coordinates in the greenhouse describing the physical locations of each plant. **(B)** Close-up schematic of a single table with the division of clover plants for the cameras. **(C)** A further close up of a picture from a camera. EW coordinates and NS coordinates for the individual pots used to identify the placement of each plant. **Box (1)** includes the raw number of tables, plants, and cameras, describing the full setup.

The system consisted of a 100Mbit network of 180 embedded computers with cameras (RPi 3 Model B with RPi Camera module version 2.1), suspended from the ceiling 2 m above 45 tables with 1,800 clover plants (**Figure 1** and **Supplementary Figures 1** and **2**). A separate internet-connected central computer was also connected to this network, centralizing the control of all 180 cameras. It received commands *via* the internet-connection to schedule picture-taking jobs that make all cameras take a picture and transfer it to the central computer. The file name of each image was annotated with a QR code read from the table. The image files were then compressed into a timestamped archive and transferred to a server outside the greenhouse for safe backup and analysis. The system was automated, provided periodical diagnostics information, and was set up to reboot and reconfigure itself after loss of electrical power. The materials and cost of the components used can be found in the system documentation (*see link in the Code Availability section*).

#### Environmental Hazards

The greenhouse can be a harsh environment for sensitive electrical equipment. Temperature and humidity varies a lot during a day cycle, with humidity being generally high. To minimize damaging effects mainly from oxidation/corrosion of exposed circuitry, precautionary measures should be taken to keep moisture from building up on the computers and camera modules. Ventilation and keeping a constant temperature will help against condensation and excess moisture. We kept the cameras running constantly so that the heat generation of 1–3 watts from their components would function as built-in radiators within their RPi plastic enclosures (**Supplementary Figure 1B**).

#### Camera System Software

The cameras are installed with Raspbian, the most common GNU/Linux based operating system for the RPi. The central computer runs scheduled bash scripts to operate the cameras *via* SSH. It updates scheduling from data found on a specified internet address, enabling remote control. When installed and configured, the system should require no user operation other than remote control. If a power cut occurs, the system will power on, self-repair file systems, and resume a productive state without human intervention.

#### Camera System Scale

The system can be grown or shrunk in size with minimal adaptation. However, the picture taking script has not been tested beyond 180 cameras. More cameras inevitably mean more physical maintenance and more points of failure as time progresses. Going beyond 250 cameras, depending on the image transfer size, additional steps should be taken to provide adequate network bandwidth availability. Transfers should be segmented into time slots or be of limited size to minimize congestion. Heterogenous scalability is possible, as newer or cheaper components become available.

#### Camera System Operation

The system used RPi 3 Model B computers with RPi Camera module version 2.1. These computers have the specification to support video streaming from the camera over a network, so getting still images was no problem performance wise.

The storage medium for RPi 3 Model B (a 16 GB MicroSD flash card) had to be acquired separately. Unfortunately, these cards have limitations in durability inherent to the current state of the technology. MicroSD cards (flash memory) are prone to data integrity loss and reduced function or failure when many write/erase operations occur to the medium or when subject to unexpected power loss. The conditions in a greenhouse environment increase the frequency of these errors. We recommend that you buy the MicroSDs from a reputable source. For further information, look in the systems guide (*see link in the Code Availability section*).

We acquired power supplies rated for a sufficient amperage to supply the RPi 3 B (2.5 A) and short USB power supply cables to prevent a drop in voltage and subsequent loss of current. During the testing phase, using power supplies with a lower specification resulted in data corruption and system failure.

#### Camera System Data

Images were taken by the RPi cameras with settings “–nopreview -w 3280 -h 2464 -q 12 -e jpg.” After image acquisition, they were transferred to the central computer and deleted from the local storage on the RPi cameras. Storing the images on a single medium in the greenhouse would pose the risk of losing all the images, so we moved the images off-site following acquisition. The images were transferred over a mobile broadband connection into a data center for stable storage and backup. Additionally, periodic backups were performed of images on-site as they were compressed into zip archives. These periodic backups were performed by a separate image taking schedule, which would run automatically and only be interrupted by the main scheduled events. The zip compressed archives yielded a compression ratio of about 12% to reduce the time required for transferring the archives over the mobile broadband, which had a weak outgoing connectivity from within the greenhouse.

#### Automatic Report System

The camera system was fully automated and designed to be remote controlled, which reduced the need for manual operation. Due to the system being fully automated, error reporting and off-site monitoring was essential. In case of problems occurring in the phenotyping system, we developed a system to report inconsistencies and reduce the amount of physical presence needed to diagnose and prevent failures. This system is divided into two parts.

Firstly, diagnostics data was periodically sent to the off-site cluster from the greenhouse. The data included timestamps and the status of the connections to all cameras. Most essential was reporting whether the expected amount of images were taken and transferred to the computing cluster in the expected timeframe.

Secondly, the off-site cluster produced a report webpage including all of the received information to evaluate problems in the phenotyping system. It also provided an email-warning, in case of failures needing immediate attention, such as loss of contact to the greenhouse server or if the schedule was not being met. Here is a list of the parameters provided for web-based view:

- Amount of images taken per camera each day
- Camera status (checking whether every camera was responding to the network test)
- Integrated image analysis pipeline information
- The ability of the pipeline to process images
- QR code detection

The reports were automatically generated and uploaded to GitHub every day and were checked frequently to monitor incoming data.

#### Pot Setup and Design

Each camera was placed so that they covered 10 pots in the greenhouse. The pots were arranged in two rows and each image captured five columns, containing 10 pots in total (**Figure 1**). The pots had dark plates underneath to allow the spread of the plants to be captured by the camera, and to provide contrast between the plates and the plants. In total, there were 45 tables, each with four cameras mounted above, tallying 180 cameras.

To conserve space in the greenhouse, the tables were moveable to allow for passage and work in between them (**Supplementary Figure 3**). During the course of the experiment the tables were moved, while work was done in the greenhouse. Due to the moveable tables, the images could not be guaranteed to have the plants located in the same place over time. Some movement of a few cameras was also observed and corrected during the course of the experiment. The unstable locations of the plants meant that the plant detection methods had to be flexible. We added QR codes to each group of 10 plants to make identification of these groups easier (**Figure 1**). All the pots were arranged in such a way that the groups were clearly visible to each camera (**Figure 1C**).

### Image Analysis Pipeline

#### Plant Location Detection Using Deep Learning

The previously detected positions of plants were not useful for plant tracking because plants could move over time (**Supplementary Figure 3**). Providing a list of fixed pot positions for each image was therefore not possible. Attempts of clustering to find each individual pot on the images proved to be unreliable due to the unequal size of individual neighbouring plants. The plants have pronounced differences in growth patterns with some growing very densely and others very dispersed. Clustering methods had a tendency of splitting individual plants into two separate clusters while joining other plants into the same cluster depending on their growth pattern or overlap (**Supplementary Figure 4**).

To help identify the group of plants desired on the images, QR codes were placed. The QR codes were placed in the center of the groups, and the pots typically were placed in the same way relative to the QR codes. A method based on using the QR code location while including the fixed positions in respect to the QR code was attempted. Using clustering methods locally at each expected position seemed to fix the main problems of the clustering methods but was very dependent on the location of the QR codes. If the QR code was not centered properly, then the fixed markers were skewed with erroneous detection as a result (**Supplementary Figure 5A**). Visibility of the QR codes either from overgrowth of the surrounding plants or from objects obscuring the QR code could also prevent correct detection of the plants (**Supplementary Figures 5B, C**).

**Figure 2:**
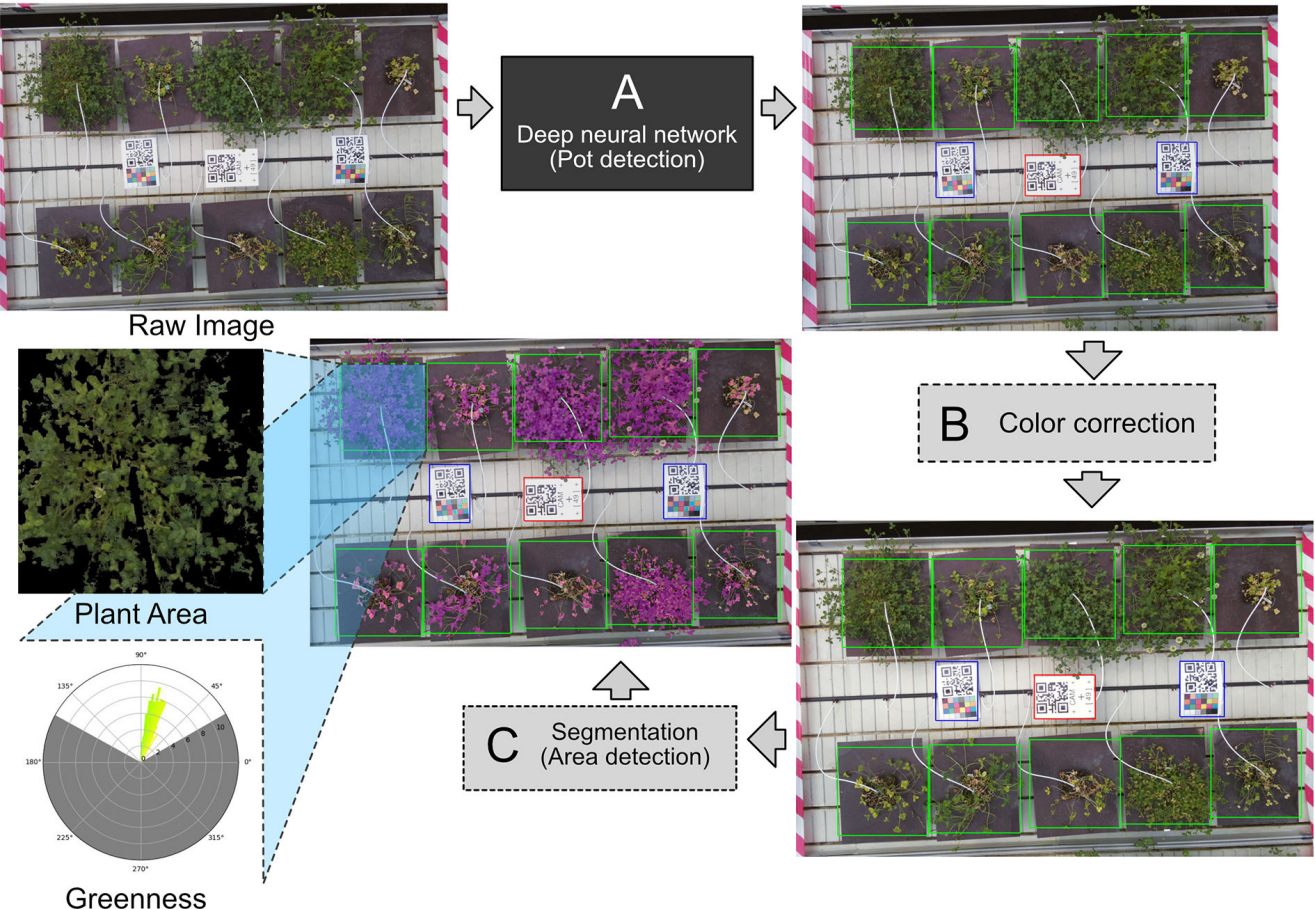
**(A)** The raw image goes through the deep neural network, which detects the pots on the image. **(B)** Color correction is applied to the image, using white balance based on the white of the center QR code. **(C)** When the pots have been identified, a segmentation algorithm is applied to the image to get area measurements of the plants. Each individual pot is cropped from the image. The plant area and greenness are measured in the cropped image. The greenness is displayed as a circular histogram. The individual crops between the pots are identical in size, making the measurement of area comparable between pots.

For a more reliable detection method, we looked to TensorFlow Object Detection API, designed to locate and find objects in an image and classify them (Huang et al., 2017). The API was ideal to solve the problem of detecting the locations of the plants. The object detection API is flexible to use, featuring several deep convolutional neural networks. The network we chose was “faster r-cnn inception resnet v2,” which has been shown to have the highest accuracy (Huang et al., 2017). The object detection was included as the first step in the image analysis pipeline (**Figure 2A**) to determine the exact location of each plant.

To train the neural network on the image setup, a visually diverse set of images capturing the diversity of the full dataset was divided into a training dataset and test dataset. The training and testing dataset consisted of 51 and 14 images, respectively. The images were selected from 10 time points with 7–10 days intervals across all cameras. We included cases where some of the pots were empty, making the plant/plate combination of the non-empty pots the target. Even if all of the plants had not been potted yet, the existing plants in the image could still be correctly identified. The training and test image datasets, which were chosen, can be seen on the Greenotyper github (https://github.com/MarniTausen/Greenotyper).

The images were labeled and classified using the tool Labelimg (Tzutalin, 2018). Three classes were defined: the first class was the POT, which should cover all of the plants and plates, the second class was the Positional QR code (QRCode), and the third was QR with ColorChecker chart (QRColor) (**Supplementary Figure 6**). The POT class was made to specifically identify the plates underneath the plants due to their clear edges. The data was trained using the provided training and evaluation scripts in the object detection API; specific pipeline settings can be found in data section training data. The training ran with 50,000 iterations and was run on a NVIDIA Tesla V100 16GB GPU using 1 GPU core, which took approximately 3 h to complete. Tensorboard (https://www.tensorflow.org/tensorboard) was used to extract the evaluated bounding accuracy of classes and the convergence of the classes. The network was exported as a frozen inference graph, which could then be used in TensorFlow for inference on newly introduced images.

The object detection was used to find all potential plants on the image, which could be more than the expected amount of 10. The QR code was used to check whether the correct group was found. All potential rows of plants were permuted into groups of 10 plants. For each group, it was checked whether they contained the QR code and the most likely group was kept. Afterwards, the group of 10 plants could be sorted and identified.

#### Organization and Pot Identification

Keeping track of each individual sample in a phenotyping system is important. To reduce the amount of bias due to environmental factors, all of the pots were randomly placed in the greenhouse. In our experiment, there were 1,800 pots to keep track of and identify. Each pot was given a unique barcode name, which could be identified using specific coordinates in the greenhouse. We used a NS (North-South) and EW (East-West) coordinate system as illustrated in **Figure 1A**. For each coordinate in the NS and EW space, we had 1 pot. Each camera contained a block of 10 NS and EW coordinates. Two files were called camera map, and ID map was created. The ID map contained the unique barcodes and positional NS and EW coordinates for the pot. The camera map contained every camera ID name tied to a specific block of NS and EW coordinates, and in which orientation the camera was relative to the NS and EW coordinate space. This relationship is demonstrated on **Supplementary Figure 7**.

The camera IDs, based on the IP address, were stored in the filename of the images produced by the camera. Each filename also contained a timestamp of when the image was taken using the following format: “*MT%Y%m%d%H%M%S*”, (*%Y: year, %m: month, %d: day, %H: hour, %M: minute, %S second*). The format of the timestamp ensured that the images were sorted in chronological order. The camera IDs from the images were extracted from the filename and looked up in the camera map to get the NS and EW coordinate ranges. The plants were then labeled and ordered with the barcodes based on the given orientation.

#### Color Correction

Light intensity and brightness changes from day to day and throughout the day. To minimize the effects of this natural variation on the data output, the images had to be color corrected. ColorChecker charts were added as a possible aid in the color correction process. These unfortunately proved to be unreliable, since the lamination on the ColorChecker charts used was reflective in natural day light obscuring the colors. Furthermore, the color was shown to fade due to the UV exposure in the greenhouse, resulting in differences in color correction over time.

Color correction requires a reference point; in this case, simply using the QR codes as a color reference seemed sufficient. The white background allows for the QR code to be used as a white color reference. White balancing is therefore possible by estimating what the value of white color is and then stretching the color channels to correct the white balance. The color correction is not as precise, as it would have been if a proper ColorChecker chart was used, but the color correction across the cameras is uniform. The same can be done using a black reference; however, the black color on the QR code is unreliable due to reflective lamination on the QR code. Demonstration of the white balancing is shown in **Supplementary Figure 8**. Color correction was applied after the plants had been found using the object detection API as the second step in the pipeline (**Figure 2B**).

### Plant Segmentation Using Thresholding and Deep Learning

Segmentation is necessary when measuring the area of the plant on the individual images. For this, we tried a traditional approach using thresholding to define masks and a deep learning approach. The main benefit of a thresholding approach over a deep learning approach is the running time. Thresholding defines thresholds for what is accepted as a “plant” on the image. For better accuracy, thresholding was done on both the HSV and CIELAB color spaces. The masks from both of the color spaces were joined, and if both masks agreed on a pixel, then this was regarded as a plant. The thresholds were adjusted by eye and were tuned to best find what were considered green/yellow pixels resembling live tissue. The CIELAB color space lends itself very well to filtering down to green/yellow pixels. The color system divides the colors into three values, L for luminosity, a* for the green-red component, and b* for the blue-yellow component. Both a* and b* color spaces range from -128 to 128. Thresholds were set to -128 to -4 and 4 to 128 for the a* and b* color spaces, respectively, keeping only green and yellow pixels and the mixture of the two.

The HSV (hue saturation value) color space is a cylinder shaped color space. Hue is the radial slice described in angular dimensions from 0° to 360° (0°, red; 120°, green; 240°, blue). Value is the vertical dimension, which describes the brightness of the color, with 0 corresponding to black and 1 corresponding to full color. Saturation is the horizontal dimension from the center of the cylinder to the sides of the cylinder, with the center of the cylinder being colorless and the sides corresponding to the color of the hue. We defined the HSV thresholds from 30° to 150° in the Hue component, capturing yellow and green colors without going into orange and blue colors. The saturation and value components were set to 0.2 to 1, excluding pale gray and dark colors. Thresholding alone is only applicable to image analysis when the surrounding setup does not include any colors, which are similar to the plants.

Instead of defining strict thresholds in HSV or CIELAB, we define slightly less strict thresholds. The problems introduced in the HSV threshold were not detected in the CIELAB threshold and vice versa. These thresholds therefore cancel each other out, leaving only certain pixels (**Supplementary Figure 9**). The area detection was included as a final step in producing the projected area measurement of the detected plants (**Figure 2C**).

Fifity ground truth masks were produced using images of both small and large plants, with different colors and light conditions to capture as much of the variation in the dataset as possible. The ground truth masks could be used in a deep learning approach to improve the segmentation over the simple thresholding method. The U-net method was chosen due to its high applicability on a small set of training data (Ronneberger et al., 2015).

The U-net was implemented using Keras (Chollet et al., 2015) in python using the TensorFlow backend, and the architecture of the model requires the resolution of the image to be a multiple of 2. The ground truth examples were split into 40 training dataset and 10 testing images. The training dataset was further split with a 20% validation split into training (32) and validation (8). To increase the size of the datasets, augmentations could be applied. As the convolutional layers are very particular about the edges they can find, rotating or moving the object on the image will be treated as a new case. Augmentations performed were not random to avoid exact repeats. The augmentations performed were flipped and not flipped, rotated (0°, 90°, 180°, and 270°) and cropped from each of the four corners with size of 460 × 460 and rescaled to 512 × 512. This increased the datasets by 40 times, training with 1,280 images and validating with 320 images. The architecture of U-net requires that the resolution images passed through the network are a multiple of 2; therefore, we used the crop size of 512 × 512. Training was done using 30 epochs using a NVIDIA Tesla V100 16GB GPU using 1 GPU core, which took around 1 h to complete. Inference of the U-net on a single cropped image is slow when using CPUs, taking around 2 s; therefore, we highly recommend running U-net using a GPU, which reduces the running time 100-fold.

#### Segmentation Accuracy

Segmentation accuracy could be assessed using the ground truth test dataset of 10 images. Different accuracy measures were applied. When comparing between the predicted mask and the ground truth, we can define true positives (TP), false positives (FP), true negatives (TN), and false negatives (FN). The Jaccard index, commonly known as the intersection over union (IoU), is a strict measure to compare the overlap between predicted mask and ground truth. It can be estimated by $\frac{TP}{TP+FP+FN}$. The Sørensen-Dice coefficient measures similarity between the ground truth and predicted masks, which can be calculated by $\frac{2TP}{2(TP+FP+FN)}$. The pixel accuracy can be estimated by $\frac{TP+TN}{TP+TN+FP+FN}$. Precision, which estimates how large a fraction of the predicted mask is correct, is calculated using $\frac{TP}{TP+FP}$. Recall, which finds how much of the ground truth was found by the prediction, is calculated by $\frac{TP}{TP+FN}$. The F1 score was calculated using the precision and recall, $\frac{2(precision\cdot recall)}{preecision+recall}$. The PASCAL VOC challenge described an average precision (AP) over recall measurements (Everingham et al., 2010). AP is estimated by calculating the area under the curve (AUC) from the maximum precision for increasing recall. Precision and Recall have an inverse relationship. You obtain higher precision with low recall or low precision with high recall. The measure is filtered for cases with IoU less than 0.5 for the AP@0.5IoU measure. This measurement was also used as the bounding box accuracy for the object detection.

#### Greenness Measure

Greenness measurements can be used to assess the state of the plant. When white clover is grown without a nitrogen source, the plants will turn yellow due to nitrogen starvation (Carter and Knapp, 2001). With the introduction of a symbiont biological nitrogen fixation is established, and the white clover will start to turn green and grow again (Sloger, 1969).

The color (hue) of the plant is captured by the hue channel in the HSV colorspace. The hue of tomato seedlings have previously been found to correlate with nitrogen content (Mata-Donjuan et al., 2012). The change in hue can be visualized using circular histograms of the hue channel. To visualize the greenness of the plants, we used the hues of the pixels labeled by the thresholding. The circular histograms clearly indicate if there is a change in color going from one hue distribution to another (**Figure 5G**). However, proper estimation of nitrogen content using the hue must be validated using true nitrogen content measurements.

To provide a quantitative measure of greenness, we take the mean and variance of the hue distribution of the plant area. A change in the mean over time indicates a change in the color of the plant. The hue channel is sensitive to the light conditions of the images, making the data noisy. With proper color correction, the hue can become less noisy and a more precise measure of greenness. In our case, using only white balancing, there was still quite a lot of noise in the data, but the signal could still be captured. To reduce the effect of the noise, we took a rolling mean with a three day window of the means in the window.

The experimental setup was focused on the effectiveness of nitrogen fixation, where each plant is dependent on the nitrogen provided from the rhizobia symbiont. To assess this quantitatively, we created a measure for quantifying the rate at which the symbiotic nitrogen fixation is established. We fitted a linear regression on the first 20 days after inoculation, when the change in color is expected to occur. The slope of the regression was defined as the RateOfHue, which was used to compare the rate at which the hue changed. A rate (slope) of zero or negative values implies that effective symbiotic nitrogen fixation has not been established and/or the plant would be dying. Positive rates imply that effective symbiotic nitrogen fixation has been established and the plant has changed color as a result. The higher the positive value of the rate, the faster the nitrogen fixation has been initiated. The estimated rate depends on the starting conditions of the plant. The possible values of the slopes depend on the differences between the starting conditions and end conditions. The rates can be corrected by the starting conditions, or they can be corrected using the starting conditions as fixed effects when modelling in linear mixed models.

#### Greenotyper Software

The image analysis pipeline has been integrated into a tool named Greenotyper. Greenotyper is both available as a command line tool and as a program with a graphical user interface (GUI). The interface is designed to make the setup of an experiment easier and test whether the pipeline works on the setup. There is a dedicated pipeline planner interface designed to make setting up the experiment/pipeline flexible and easy to use (**Supplementary Figure 10**). The pipeline planner allows the user to test the image analysis pipeline with their own images and customize the pipeline to their own experiment. The Greenotyper pipeline process begins with taking the raw image and applying the neural net model to the image to identify all of the plants. Color correction is then applied to the image if applicable. Plant area detection is then applied to the image and the measurements are taken for each individual plant. The Greenotyper pipeline is illustrated in **Figure 2**.

Greenotyper can provide four kinds of outputs: the cropped image, the masked image used in estimating plant area, the projected plant area, and the measure of greenness for each plant. The output files can be subdivided into directories based on time of capture or by sample/individual ID. The plant area estimates and measure of greenness are written into their own respective files.

To enable scalability of the pipeline, multithreading/multiprocessing is implemented in Greenotyper. In the GUI, multithreading is handled by the PyQt library, while the command line interface uses the standard library multiprocessing, specifically the pool command. Multiprocessing enables the use of the full capacity of cores available in the computer. For large scale analysis, like the one performed here, running the analysis on a large computing cluster can significantly reduce the running time. File locking was used to ensure that the plant area, and greenness data were not concurrently written to the file; therefore, none of the data was lost or corrupted.

**Figure 3:**
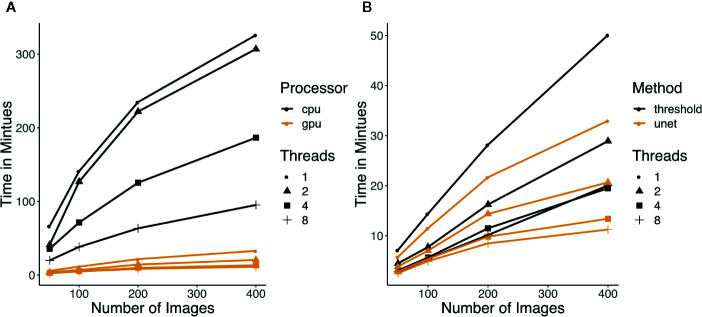
**(A)** Running time of U-net using CPU versus GPU. Color indicates which processor was used and the shapes of the points represent how many threads were used for the running time measurement. **(B)** Running time of thresholding and U-net (GPU) with increasing number of images. Each run was done with 3 replicates and the median was taken. The colors represent which method was used and the shapes of the points represent how many threads were used for the running time measurement.

When using U-net for segmentation in Greenotyper, the pipeline was divided into three parts. First, object detection and color correction are applied as a preprocessing step saving the images as numpy matrices (.npy) on disk. Afterwards the U-net is applied, where this step can be run using the GPU to reduce the running time 100 times (**Figure 3A**). The last step is a postprocessing step using the masks produced by the U-net and the image data to write all of the outputs to disk. Using thresholding runs everything in a single pass and runs approximately in the same time as the preprocessing and postprocessing step together.

The object detection training is not included in the Greenotyper tool; however, a guide to perform the training is found in the GitHub repository (https://github.com/MarniTausen/Greenotyper). U-net training with data augmentation is implemented in the Greenotyper tool.

## Experiments

### Experimental Design

To test the genotype-genotype interactions of white clover (*Trifolium repens*) and its symbiont *Rhizobium leguminosarum* symbiovar *trifoliii (Rlt)*, 148 clover genotypes were grown (Griffiths et al., 2019) in a binary setup with one of 170 natural genetically characterized Rhizobium isolates (Cavassim et al., 2020). A total of 3,600 plants were grown under nitrogen limited conditions in two rounds in irradiated peat (round 1, 1,800 plants) and vermiculite (round 2, 1,800 plants). Clover genotypes were propagated from cuttings produced in a separate greenhouse to ensure the replicates were genetically homogenous. No nitrogen was applied in the fertilizer. There were two to three replicates of each clover-rhizobium combination grown per round, which had been randomly selected. Ten plants were used as uninoculated controls. The plants were harvested after a growth period of 42 to 52 days for round 1 and 68 to 70 days for round 2. Genotype-genotype interactions, genomic prediction, and genome wide association studies will be explored in detail in a future manuscript.

### Growth Measurements

The output data can be formatted and sorted by Greenotyper, with each individual sample in a column and the recorded time points in rows. The data for each individual plant can be converted into a growth curve by averaging the size of the daily measurements. There is variation in the plant size estimates throughout each day, and to avoid the effects of strong outliers, we used the median instead of the mean. The variance per day was used to filter out days with unusually high variance by eliminating all points with variance greater than three times the standard deviation.

The area under the growth curve was standardized by subtracting the initial size of the plant, which varied greatly between genotypes since plants were grown from cuttings and not seeds. AreaPerDay was calculated by dividing the standardized area under the growth curve by the growth period in days. To normalize AreaPerDay, the standardized area under the growth curve was calculated in a 30-day window for all samples.

### Heritability Estimates

Heritability estimates for AreaPerDay and RateOfHue (rate of change in greenness over time) were calculated to test whether some of the variation measured could be explained by the genotypes in the experiment. The heritability is calculated from the variance estimates from the fitted linear mixed models. For the estimation, we used the lme4 R package and the lmer function (Bates et al., 2015). Twenty-two plants were manually filtered out due to abnormal growth patterns, and all controls labeled “NO” (uninoculated plants) were filtered out. Plants inoculated with a confirmed contaminated rhizobium strain were also filtered out. The AreaPerDay was corrected using the initial size of the plants. The model estimation for AreaPerDay was run as follows, with only clover and Rhizobium as random effects:

$$\begin{array}{l} \text{lmer}\;(\text{AreaPerDay}\;\;∼\;\text{NS}\;+\;\text{EW}\;+\text{Round}\;+\;\text{Replicate}\;+\\ \left(1|\text{Clover})\;+\;(1|\text{Rhizobium})\;,\;\text{data}=\text{growth}\_\text{data}\right) \end{array}$$

This accounts for the variation of location (NS and EW coordinates), period of growth round, and replicate information. RateOfHue was estimated using the following model:

$$\begin{array}{l} \text{lmer}\;(\text{RateOfHue}\;\;∼\;\text{NS}\;+\;\text{EW}\;+\text{Round}\;+\;\text{Replicate}\;+\\ \left(1|\text{Clover})\;+\;(1|\text{Rhizobium})\;+\;\text{StartHue},\;\text{data}=\text{growth}\_\text{data}\right) \end{array}$$

The RateOfHue includes the StartHue, which is strongly correlated.

The broad sense heritabilities for Clover would be estimated as: (*var_clover_*)*/sum*(*var*). In this case, *sum*(*var*) would be estimated as (*var_clover_*+*var_rhizobium_*+*var_residual_*). These estimates do not correspond to the narrow sense heritability, the amount explained by genetic information (SNP/variant information), instead the broad sense heritability is the maximum heritability that can be estimated since it captures the total amount of variation that can be explained by the genotype information including genotype × environment effects.

### Running Time Experiments

Running time tests were performed using thresholding and using U-net. The U-net runs were divided into two categories using either CPU or GPU for the U-net. All running time experiments were run using the same approach. Samples sizes were 50, 100, 200, and 400 images, and each were run with one, two, four, and eight threads. Each was run with three replicates, and the median running time was used. All of the CPU tests were run on a 2015 mid Macbook Pro with a 2.2 GHz Intel Core i7 processor. The GPU tests were run on a NVIDIA Tesla V100 16GB GPU.

## Results

### Camera System and Image Acquisition

The experiment was carried out over 146 days in two separate rounds, during which the camera system was active. There was a 26 day break between the two rounds, where the camera system was still active, but the images acquired during this period were not used in the analysis. The camera system was configured to take images within the time interval 10.00 to 17.00 every half hour (14 images per day). The success rate of the camera system across all cameras, with and without backup, is summarized in **Table 1**. The expected number of images was calculated by taking the days of operation (146) times the number of images taken per day (14). For a single camera, the resulting expected value was 2,044 images, and for all cameras, it was 367,920. The total uptime was calculated by comparing the number of expected images to the number of images included in the final analysis. *Without backup* represents the number of images taken and successfully transferred over the network to the computer cluster for storage (316,790). *With backup* includes this number plus all images recovered from the autonomous backup routine (355,027). Without backup, the average camera had an uptime of 86% compared to an uptime of 96% with backup. Without the backup 10% of the data would have been lost due to connection issues between the greenhouse and the storage cluster, and the remaining 4% of the data loss was due to internal problems in the camera system. Without the backup, 161 out of 180 cameras had an uptime of approximately 85%. Including backup, 168 of the cameras had an uptime above 95% (**Supplementary table 1**).

**Table 1:** Summary of the stability of the phenotyping camera system.

	Transferred images *without backup*			
	**All Cameras**	Average camera	Worst camera	Best camera
Operation time (days)	146	146	146	146
Uptime (%)	86%	86%	78%	89%
Pictures	316,790	1,759.94	1,591	1,815
Space usage	346 GB	1.97 GB	1.6 GB	2.2 GB
Expected amount pictures	367,920	2,044		
	**Transferred images *with backup***			
	**All cameras**	**Average camera**	**Worst camera**	**Best camera**
Operation time (days)	146	146	146	146
Uptime (%)	96%	96%	88%	99%
Pictures	355,027	1,972.37	1,803	2,027
Space usage	380.4 GB	2.11 GB	1.7 GB	2.4 GB
Expected amount pictures	367,920	2,044		

The operation time is the amount of days the experiment was running. Uptime was calculated based on the camera’s ability to produce images, and the percentage is the fraction of images taken in the experiment divided by the expected amount of pictures given the time frame. Pictures: The number of pictures taken. Space usage is the total storage space usage of the images. Expected amount of pictures is the amount of images we expected based on the length of the experiment. The tables have been subdivided into two groups, without backup and with backup. Without backup represents the number of images taken and successfully transferred to the main cluster for storage. The backup included all images taken at the greenhouse which later were added into the main storage.

### Object Detection Bounding Box Accuracies

The neural net was trained with 51 training images and 14 evaluation images. Each image contained at least 10 pots and three QRcodes, meaning that it included several instances of each class (**Table 2**). Bounding box accuracy was estimated as the AP over recall or PASCAL VOC AP@0.5IoU (Everingham et al., 2010), which evaluates how precisely the network can reproduce bounding boxes from the testing dataset. The bounding box accuracies for three different models are shown in **Table 2**. Model clover_v1 only contained one class, POT, which was used for the plants/plates. The bounding box accuracy for POT in clover_v1 was 97%. Model clover_v2, which included the classes POT, QRColor, and QRCode, had the bounding box accuracies 97.80, 97.20, and 100%, respectively, improving slightly on the POT accuracy from clover_v1. The clover_v3 model was further run on the clover_v2 adding 50,000 extra steps. Running it with more steps did not improve much on the accuracies, resulting in 97.98% Pot, 97.27% QRColor, and 100% QRCode, showing that 50,000 steps are sufficient to reach good accuracies.

**Table 2:** Bounding box accuracy (PASCAL VOC AP@0.5IoU Everingham et al., 2010) measured on 14 testing images.

Dataset	Images	Pots	QR codes	QRColor
Training	51	510	51	102
Evaluation	14	140	14	28
**Model/class**	**POT**	**QRColor**	**QRCode**	**Total Steps**
clover_v1	97%			50,000
clover_v2	97.80%	97.20%	100%	100,000
clover_v3	97.98%	97.27%	100%	150,000

The accuracy is shown for two versions of the model. Clover_v1 only contained the class POT, while clover_2 and clover_v3 contained the classes POT, QRColor and QRCode.

### Running Time of Greenotyper

The running time of Greenotyper increases linearly with the increase in the number of input-images, and the speed can be improved with the use of more threads or processes (**Figure 3**). U-net is highly dependent on access to a GPU for reasonable running times (**Figure 3A**). Running times using U-net on a CPU are much higher than on a GPU, spending 2 s versus 20–30ms per cropped image. The running time on a CPU is highly unstable and depends on the file system, because reading and writing temporary files to conserve memory are required. Using the U-net on a GPU accelerates the prediction, with 100 images taking around 5 min using a GPU and taking around 38 min using a CPU, both using eight threads (**Figure 3A**). Running U-net on a GPU also runs faster than using thresholding (**Figure 3B**). Increasing the number of threads or cores that Greenotyper uses reduces running time in all cases (**Figures 3A, B**).

Using a large computing cluster to distribute the images into batches allowed the processing of all 355,027 images to be run in approximately 24–36 h. The computing cluster GenomeDK (genome.au.dk) allowed for 100–200 images to be processed simultaneously. To put the scale and size into perspective, analyzing all of the images on a single quad-core i7 processor with four to eight threads would take approximately 2 weeks. Running U-net on all images using GPU processing when applying the U-net could also be completed in approximately 24–36 h. Running U-net on large-scale data using CPUs would be expected to result in an approximately 100-fold increase in running time.

### Segmentation Accuracy

Segmentation accuracies for the thresholding method and U-net were assessed on 10 testing images from the ground truth dataset. The thresholding method was split into three categories, HSV, LAB, and HSV/LAB, with the thresholds on their own and the combined thresholds (**Table 3**). The HSV threshold alone showed the poorest performance with low precision (0.66), Jaccard index (0.65), Sørensen-Dice coefficient (0.76), and pixel accuracy (0.89). The recall was very high (0.97), which means that there are very few false negatives (**Table 3**). The LAB threshold alone performed better with a much higher precision (0.8). However, the combined HSV/LAB threshold performed better in nearly all categories, with the exception of the recall (**Table 3**).

**Table 3:** Segmentation accuracy of the thresholding methods (HSV only, LAB only, and combined HSV and LAB) and the U-net method and finally the difference between HSV/LAB and U-net.

	HSV	LAB	HSV/LAB	U-net	Diff
**Jaccard index (IOU)**	0.65	0.75	0.77	0.84	+0.07
**Sørensen-Dice coefficient**	0.76	0.85	0.86	0.91	+0.05
**Pixel accuracy**	0.89	0.93	0.93	0.95	+0.02
**Precision**	0.66	0.80	0.83	0.89	+0.06
**Recall**	0.97	0.93	0.91	0.93	+0.02
**AP [0.5 IoU]**	0.91	0.93	0.94	0.96	+0.02
**F1 score**	0.78	0.86	0.87	0.91	+0.04

Segmentation accuracy measures, Jaccard index or intersection over union (IoU), the Sørensen-Dice coefficient, pixel accuracy, precision, recall, F1 score estimated from the precision, and recall and AP for predictions over 0.5 IoU as described by the PASCAL VOC challenge (Everingham et al., 2010).

The U-net performed very well with a high Jaccard index (0.84), Sørensen-Dice coefficient (0.91), pixel accuracy (0.95) (**Table 3**). The U-net had the highest precision (0.89), finding fewer false positives while still having high recall (0.93). The AP over recall was the highest among all of the methods (0.96). The largest increase was in the Jaccard index or the intersection over union (IoU), with an increase from 0.77 when using HSV/LAB to 0.84 when using U-net.

**Figure 4:**
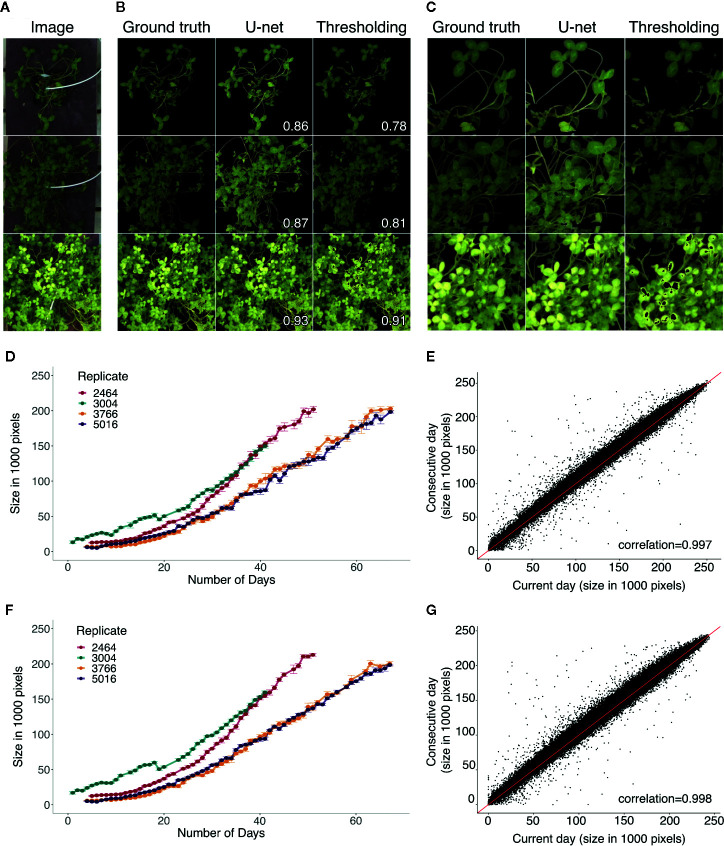
**(A)** Three example images from the test set for segmentation. **(B)** Masks produced to the corresponding examples from **(A)**, showing the ground truth mask and the predictions from U-net and thresholding. Corresponding Jaccard index (intersection over union) statistics between the ground truth and predicted masks are displayed. **(C)** Zoomed in examples on the masks showing regions in which the U-net improved the prediction compared to thresholding. **(D, F)** Growth curve examples from the same replicates from the two different rounds, with replicates 2,464 and 3,004 from round 1 and replicates 3,766 and 5,016 from round 2. Growth curves made using thresholding **(D)** and U-net **(F)**. The units are the size of the plant area measured in the number of pixels from the predicted masks. **(E, G)** Change in plant size from the current day to the consecutive day across all days and plants using thresholding **(E)** and using U-net **(G)**. The red line corresponds to a 1:1 relationship. Pearson correlations are shown on bottom right corners **(E, G)**.

U-net detects the plants more accurately than thresholding, and is capable of detecting the stems where thresholding struggles (**Figures 4A–C**). The segmentation using thresholding is also inaccurate in cases where the plants were hit directly by sunlight, which occurred during a limited time period on sunny days (**Figures 4A–C**).

### Growth Measurements

Visual examples of growth curves produced for the same four replicates from two rounds are shown on **Figures 4D, F**. Thresholding (**Figures 4D, F**) and U-net (**Figures 4F, G**) produced consistent results. The AreaPerDay measurements estimated from the growth curves were highly consistent between U-net and thresholding, with a pearson correlation of 0.983 (**Supplementary Figure 11A**). Comparing from 1 day to its consecutive day across all growth curves showed stable plant size measurements over time (**Figures 4E, G**). Most of the change in size to the following day is an increase in size, with few cases deviating with a small decrease and a small number of outliers with large variation (**Figures 4E, G**). The daily variation in area measurements were mostly due to the movement of the plants throughout the day (**Supplementary Figure 12A**). The distribution of the variation of the size per day generally shows low variation 1,500 pixels with a mean of 2,500 (**Supplementary Figure 12B**). The CVs show that for small plants (*<*20,000 pixels), the variation is higher (**Supplementary Figures 12C, D**). For images measuring plants with very small sizes, there is a large amount of variation, likely caused by the noise of the segmentation. U-net has lower variation for small plants than thresholding, suggesting less noise (**Supplementary figures 12C, D**). Data from days that showed variation more than three standard deviations from the mean were filtered out as they were deemed too noisy to include.

### Greenness Measurements

**Figure 5:**
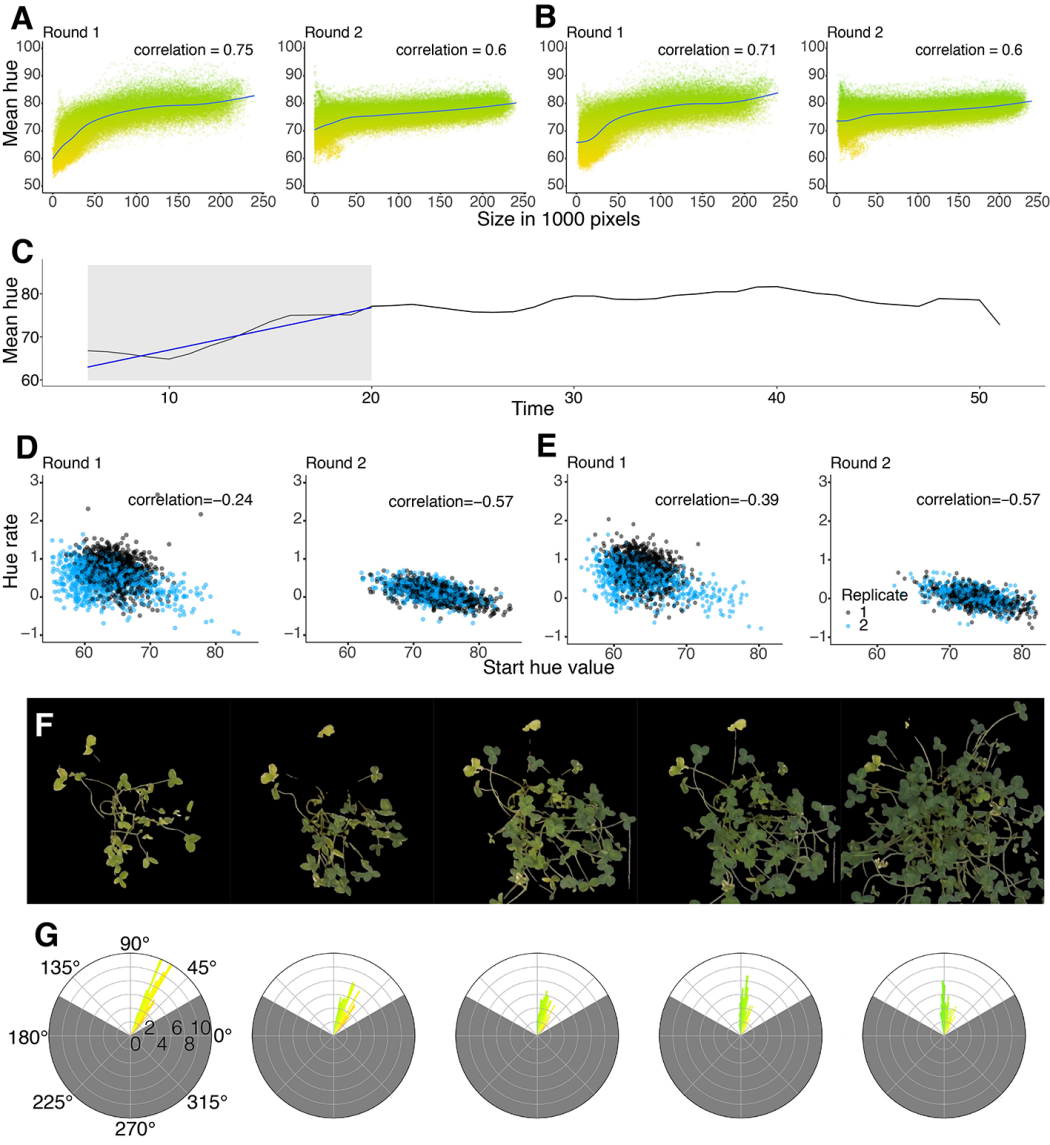
**(A, B)** Correlation between the mean of the hue and the size of the plant. Y-axis shows the hue ranges from Yellow (50°) to Green (100°). All time points across all plants are included on the figure. Comparison between Thresholding **(A)** and U-net **(B)** showing the difference between round 1 and round 2 for each method. **(C)** Example of how the RateOfHue was estimated. The grey area corresponds to the mean hue values fit with a linear regression. The regression curve is drawn with a blue line. The rate of hue corresponds to the slope of the regression. **(D, E)** Pearson correlations between the rate of hue and the starting hue value of round 1 and round 2 for Thresholding **(D)** and U-net **(E)** across all plants. **(D)** Round 1 has a -0.24 Pearson correlation and round 2 has a -0.57 Pearson correlation. **(E)** Round 1 has a -0.39 Pearson correlation and round 2 has a -0.57 Pearson correlation. **(F)** Graphical example of a single plant changing color over time. The masks were produced using U-net. **(G)** Circular histograms of the hue distribution from the corresponding masks above. The scales around the histogram are the degree value of the hue, and the scale within is the percentage of the count with a particular hue value. The gray area of the circular histogram corresponds to the hues filtered by the mask. The colors of the bars in the histogram indicate the hue of the pixels in the corresponding mask.

In addition to plant area, we were also interested in quantifying plant greenness, since this is an indication of the nitrogen fixation status of white clover. The experiment was run in two rounds, one after the other. The greenness measured using Thresholding (**Figure 5A**) and U-net (**Figure 5B**) were very similar, showing the same patterns and similar correlations in both rounds. Comparing the mean of the hue from round 1 to round 2, there is a clear difference between the two rounds (**Figures 5A, B**). In round 1, the plants clearly started with a more yellow hue (**Figures 5A, B**) than the round 2 plants, suggesting that the round 2 plants likely did not become as nitrogen starved prior to inoculation with rhizobia (**Figures 5A, B**). The rate of change in hue (RateOfHue) was calculated as the slope of a linear regression based on the first 20 days, starting from day 3 due to the rolling mean applied (**Figure 5C**). The RateOfHue estimates produced from U-net and thresholding were highly correlated (r = 0.948), implying that the rates estimated between the two methods are highly concordant (**Supplementary Figure 11B**). The starting hue value was negatively correlated to the rate of the hue estimates (**Figures 5D, E**). The replicates in round 2 were not different, but the second replicate from round 1 seemed to estimate lower rates than other replicates (**Figure 5D**). The most likely reason, this replicate performed worse, was that the greenhouse temperature peaked during the time when round 1 replicate 2 was set up. This would likely have stressed both the plants and rhizobia, causing them to deviate from their normal interactions and growth patterns. When using U-net, rates of the second replicates in round 1 were slightly improved (**Figure 5E**). The change in hue clearly corresponds to the change in color, and during the phase of changing color, the distribution of hues is more variable (**Figures 5F, G**).

### Heritability Estimates

To evaluate if the area and greenness traits we had derived from the image data were controlled by the clover and rhizobium genotypes, we calculated heritabilities. The heritability was calculated from the data estimated from thresholding and U-net (**Table 4**). Heritabilities estimated for both of them were similar, but U-net had slightly lower heritability in nearly all cases (**Table 4**). For AreaPerDay using thresholding, the clover broad sense heritability was 33.92%, and for rhizobium it was 3.64%, indicating that clover and rhizobium genotypes together explain 37.56% of the phenotypic variation (**Table 4**). Heritability was also estimated for the two rounds separately, which resulted in combined clover and rhizobium heritabilities of up to 55.77% (**Table 4**). RateOfHue heritability was 9.69% for clover, 5.99% for rhizobium, and 15.68% in total when all rounds were included (**Table 4**). The RateOfHue heritability was higher when the data were separated into rounds, with the second round having a combined heritability of 32.37% (**Table 4**). Removing the second replicate of round 1 increased the heritability to a combined heritability of 22.79%, and only looking at the first replicate of round 1 had the highest heritability of 37.85%. For the RateOfHue trait, the rhizobium heritability was higher than that of clover, but the combined heritability was markedly lower than for the AreaPerDay trait (**Table 4**).

**Table 4:** Broad sense heritability estimates for the measurements produced from the image data for both U-net and thresholding (HSV/LAB).

		Clover		Rhizobium		SUM	
Measure	Filter	U-net	HSV/LAB	U-net	HSV/LAB	U-net	HSV/LAB
AreaPerDay	None	33.02%	33.92%	3.71%	3.64%	36.73%	37.56%
AreaPerDay	Round 1 only	41.95%	42.39%	5.31%	4.92%	47.26%	47.30%
AreaPerDay	Round 2 only	44.67%	50.78%	1.85%	4.99%	46.52%	55.77%
AreaPerDay	No round 1 rep 2	38.60%	40.09%	1.16%	1.16%	39.76%	41.25%
RateOfHue	None	9.40%	9.69%	4.93%	5.99%	14.33%	15.68%
RateOfHue	Round 1 only	10.84%	11.37%	8.07%	8.80%	18.91%	20.17%
RateOfHue	Round 2 only	20.83%	20.13%	7.63%	12.24%	28.46%	32.37%
RateOfHue	No tound 1 rep 2	17.08%	16.95%	5.60%	5.84%	22.68%	22.79%

For AreaPerDay and RateOfHue filters, none (four replicates), round 1 only (two replicates), round 2 only (two replicates), and no round 1 rep 2 (three replicates) were applied. The models used are described in the Heritability Estimates section.

### The Greenotyper Pipeline

Out of 3,600 monitored plants, 3,562 plants were successfully included in the image data analysis using thresholding, and 3,568 plants were successfully included in the image analysis when using U-net. The remaining plants were removed because of too much missing data. The missing data was caused by the cameras/plants being moved during the course of the experiment. This meant some of the intended plants of the experiment were not on the image, which caused problems in the detection/filtration step when looking for a group of 10 plants (**Supplementary Figure 13**). However, 99.1 and 98.9% of the plants were measured when using U-net and thresholding, respectively, providing a near-complete dataset for downstream analysis.

## Discussion

### Camera System and Image Acquisition

The RPi camera system remained very stable despite a humid greenhouse environment with occasional extreme temperatures of up to 46°C. Our setup included monitoring of data transfer coupled with automatic alerts that were issued in case of issues with the greenhouse camera system. As unexpected problems can occur, we highly recommend generating a daily report, which is automatically distributed to the camera system administrators. Similar automatic reporting, in this case using Slack messaging, has previously been used to report when temperature and light conditions fall outside certain thresholds (Grindstaff et al., 2019). Our system did not require much maintenance, and maintenance could be performed outside of camera operating hours.

Recently, camera systems using RPi units have been described by Minervini et al. (2017); Tovar et al. (2018), and Grindstaff et al. (2019). Different configurations of RPis have been proposed, including overhead fixed cameras, units attached to a camera arm, and units arranged in multi-image octagons to capture three-dimensional information (Tovar et al., 2018). RPi units can be modified to allow recording of temperature, light, and humidity in addition to image data (Grindstaff et al., 2019), while Phenotiki offers camera system management and provides an image analysis pipeline (Minervini et al., 2017). These different setups were tested in growth chambers as proof of concepts, but have not been used to monitor large-scale experiments.

### Neural Net Training Bounding Accuracies

The neural net plant detection accuracy was very high. The training of the POT classifier was designed to focus on the plate beneath the plant. This enabled identification of the future position of the plant before potting the plant itself, facilitating registration of relative positions for plant identification.

The QR codes also proved very easy to detect. The QRCode class had an accuracy of 100%, whereas the QRColor squares were less efficiently detected because the ColorChecker QR codes were obscured by the plates in some cases. It proved sufficient to run the training for 50000 steps. Adding an additional 50,000 steps to the training provided only miniscule improvements.

Using our trained network on a different setup will likely be unsuccessful, as it will be trained at finding the particular patterns of plate and clover morphology in general. However, with LabelImg (Tzutalin, 2018) and the object detection API, training is not difficult to set up. The classification in LabelImg is done manually, while the training is then further processed in the object detection API. The running time of the training is highly dependent on access to a GPU. Running it on a CPU is possible, but the runtimes should be expected to increase significantly.

Deep learning approaches have been implemented in other phenotyping experiments. A CNN network for identifying and classifying *Arabidopsis* genotypes used a long short-term memory (LSTM) structure to include temporal information for all of the individual genotypes, allowing them to incorporate individual genotype identifiers for training (Taghavi Namin et al., 2018). How such a system would work with data from a large-scale experiment as presented here is currently unknown. Using object detection only for identifying the location of the plants makes detection simple and reliable. Identification of plant individuals is then carried out based on camera IDs and the relative locations of the plants.

### Running Time of Greenotyper

The Greenotyper pipeline can be run using simple thresholding or a deep learning-based U-net with similar running times, provided that U-net can be run on a GPU. Running the U-net analysis on a CPU is possible, but results in greatly increased running time. Running U-net using a GPU is slightly faster than thresholding on a CPU, but if access to multiple GPUs is limited, the thresholding pipeline is potentially much faster for large scale experiments. Using more threads/cores can be used to speed up the pipeline; however, the decrease in running time when using too many threads can be limited.

A major bottleneck in large scale runs is the file writing. Writing to the same file with multiple processes comes with the risk that processes can write to the same file at the same time. File writing by default is not “thread-safe”; therefore, we used file locking to keep the data intact. File locking makes the file writable by a single process at the time, while the other processes must wait until the file can be opened again. This has a major impact on the running time, as a writing time of only 0.01 s adds up to 1 s for every 100 processes in line.

Our recommended way of running a large-scale analysis of the images is using a distributed computing cluster. Being able to run 100 images at a time greatly decreases the running time. Each image can be processed as a separate process, or images can be grouped into batches of several images running under the same batch process. When running with U-net and having access to a GPU, the pipeline is divided into three steps: (1) preprocessing and object detection, (2) U-net, and (3) post-processing (results and outputs). Pre- and post-processing can be run using the full capacity of the distributed computing cluster, and the U-net step can be run on the GPU for prediction after preprocessing. If the number of GPUs is limited while CPUs are abundant, then this strategy can be used to speed up the pipeline substantially.

Phenotiki has a fast running time using 5.5 s per image (Minervini et al., 2017). Greenotyper using U-net on a GPU or using thresholding achieves comparable speeds of around 3 s per image. Training Phenotiki is substantially faster (3.5 min) than U-net (50 min) and does not require a GPU. How phenotiki performs on large scale experiments is unknown, and its parallelization capabilities are not described. An unsupervised machine learning method, ULCRF, has been described for segmentation but has longer running times of 95.25 s per image (Zhang and Xu, 2018).

### Segmentation Accuracy

The combined HSV/LAB thresholding strategy improves the segmentation accuracy over using the individual HSV or LAB thresholds. However, it is clear the majority of the accuracy of the thresholding masks comes from using the CIELAB threshold. The CIELAB threshold is very simple, choosing only the color dimensions that include green and yellow colors. The CIELAB threshold has recently also been found to provide higher accuracy in thresholding segmentation methods compared to the HSV or RGB color spaces (Riehle et al., 2020).

U-net improves the segmentation accuracy, being capable of capturing the whole plant much better than thresholding. It performs better in general by having a more precise overlap between the ground truth and the prediction than thresholding. The U-net requires quite extensive and diverse training data, and time must be spent to produce ground masks that can be used for training, validation and testing. To increase the size of the datasets, augmentations can be applied to simulate “new” data. The only requirement is that the images augmented are not too similar, which may lead to overfitting to the training data. Data augmentations have also been applied in other plant phenotyping deep learning approaches, where rotations were applied to increase the number of training images three-fold (Taghavi Namin et al., 2018).

Phenotiki uses an incremental learning approach to train a plant appearance model using past segmentations to improve the current one (Minervini et al., 2014). The method achieved very high accuracies with a Jaccard index of 0.932 and 0.964 in the Sørensen-Dice coefficient (Minervini et al., 2014). Lee et al. (2018) proposed a different approach using super pixels to enlarge regions of interest and using a random forest classifier. The method got F1 scores of around 0.795 on average on the testing data, but found a very high AP of 0.95, which was comparable to the AP of our U-net analysis of 0.96. Both methods were trained and tested on *Arabidopsis* rosette plants. How these scores would translate to the white clover is unclear, but they provide possible alternative segmentation approaches. Unsupervised approaches, which do not require training, can also be explored. The unsupervised learning method ULCRF performed fairly well with an average pixel accuracy of 0.75 in segmenting both plant and fruit of tomatoes (Zhang and Xu, 2018).

### Growth Measurements

U-net and thresholding produced very similar growth curves and plant size estimates. The growth curves were generally stable, showing gradual daily increases in plant size. The AreaPerDay measurements from U-net and thresholding were also consistent. To account for the variation each day, the median is a robust estimate, reducing the effect of any strong outliers. The variation within each day seemed largely influenced by the movement of the plants throughout the day. Previously, the movement of plants has been observed due to changing wind conditions, which added variation to the measurements each day and between days (Guo et al., 2017). The shape or area of the plants visible on the images is altered and therefore adds variation to the measurements. A few of the days had higher variation, and many of these were due to poor detection, often caused by a failure to return the mobile greenhouse tables to their original position, which resulted in a different plant being labelled and detected.

The growth curves have a tendency to show an increase and then reach a plateau towards the end. The plateau occurs when the plants have grown beyond the area measured, which corresponds to the area of the plate. The plateau can be avoided by increasing the dimensions of the crop, but in our case that also caused an increase in the likelihood of erroneously measuring the neighboring plants. Training a mask R-CNN network to detect the boundary boxes of the individual plants could remove the plateau altogether (He et al., 2018), but would possibly still fail when the plants become so large that they overlap with their neighbours.

The loss of quantitative image data from 32 to 38 out of 1,800 plants was attributed to positioning of the cameras in relation to the plants. These errors can occur as the cameras may move due to disturbances in the greenhouse or ongoing work in the greenhouse requires moving the plants. U-net was capable of capturing slightly more of the problematic cases than thresholding, including data from six additional plants.

The heritability estimates from using thresholding or U-net were largely very similar, suggesting that both methods are able to capture the same growth dynamics properly. The U-net would be preferred due to its higher accuracy of the segmentation, but if the U-net cannot be run due to the lack of a GPU, then thresholding is capable of generating nearly the same results.

Other morphological features can be extracted from the produced image masks. For instance, the images output by Greenotyper can be used to extract the leaf count using PlantCV (Gehan et al., 2017). Joining the Greenotyper pipeline with other tools thus enables the construction of new custom pipelines.

### Greenness Measurements

The mean of the hue distribution has previously been shown to correlate with laboratory measurements of plant nitrogen content (Mata-Donjuan et al., 2012). Direct estimation of nitrogen levels is not possible unless it can be calibrated using a SPAD chlorophyll meter (Turner and Jund, 1994) or in a laboratory. Other greenness measurements can be applied but may depend on the setup of the experiment. For example, comparing the hue between measurements from different time points using Euclidean distances can be used to detect early stages of macronutrient deficiencies (Wiwart et al., 2009).

Comparing the greenness levels between plants was deemed unreliable, as the variance of the hue is high unless there is a high degree of certainty in the color correction. Instead, we used relative changes in greenness, RateOfHue, as a proxy for plant nitrogen status. It proved to be a heritable trait, suggesting that it may reflect a relevant biological process, but direct comparisons to leaf nitrogen content dynamics would be required for confirmation. The hue dynamics varied across the greenhouse experiment rounds with round 2 seeming to not reach a nitrogen-starved yellow state. The heritability for the whole experiment was lower than for each round analyzed separately, suggesting that hue measurements are sensitive to the specific setup of the individual experiments.

## Conclusion

A phenotyping system based on a network of RPi computers proved to be a useful and affordable method of measuring plant growth. The image analysis pipeline provided a reliable detection method for plant area with multiple plants on the same image. The analysis was robust under the challenging conditions in the greenhouse, such as variable locations of the plants and unpredictable and overlapping growth patterns. In addition, it proved possible to quantify the rate of change of plant hue, or greenness, which could facilitate genetic mapping of plant nitrogen status and fixation. The GUI of the Greenotyper tool provides a guide for setting up an image analysis pipeline for a large and complex experiment, facilitating future large-scale studies.

## Code Availability

The software and supplementary data can be found publicly available on github.

- Phenotyping camera system manual: https://github.com/MarniTausen/CameraSystemDocumentation
- Automatic Report system scripts:
- https://github.com/MarniTausen/AutomaticReportSystem
- Greenotyper (Image analysis pipeline):
- https://github.com/MarniTausen/Greenotyper

## Data Availability Statement

All datasets generated for this study are included in the article/**Supplementary Material**.

## Author Contributions

Conceptualization: MT, MC, SM, SA. Methodology: MT, MC, AS. Software: MT. Formal Analysis: MT. Investigation: SM, MT. Writing—Original Draft: MT. Writing—Review and Editing: MT, MC, SM, SA, LJ, AS, AD. Visualization: MT, SM. Supervision: SA, LJ, AD. Project Administration: SA. Funding Acquisition: SA.

## Funding

Grant provided from Innovation Fund Denmark (grant 4105-00007A to SA).

## Conflict of Interest

The authors declare that the research was conducted in the absence of any commercial or financial relationships that could be construed as a potential conflict of interest.
