# Supplementary material for: Greenotyper: Image-Based Plant Phenotyping Using Distributed Computing and Deep Learning
Source: Front Plant Sci. 2020 Aug 7;11:1181. doi: 10.3389/fpls.2020.01181 (PMC7427585; doi:10.3389/fpls.2020.01181)
Supplement: Supplementary file 1 [file DataSheet_1.pdf]

Supplementary Material

1 SUPPLEMENTARY DATA

The data mentioned in this study are openly available at the following locations:

- All round 1 images from Camera 41 (Data from 10 pots): https://github.com/MarniTausen/Greenotyper/tree/master/sample_data/Cam41
- Network, Camera map and ID map: https://github.com/MarniTausen/Greenotyper/tree/master/sample_data
- Growth curve and Greenness measurements: https://github.com/MarniTausen/Greenotyper/tree/master/results
- Training and Testing data: https://github.com/MarniTausen/Greenotyper/tree/master/training_data

2 SUPPLEMENTARY TABLES AND FIGURES

Figure have been uploaded to Figshare: 10.6084/m9.figshare.11365973

2.1 Figures

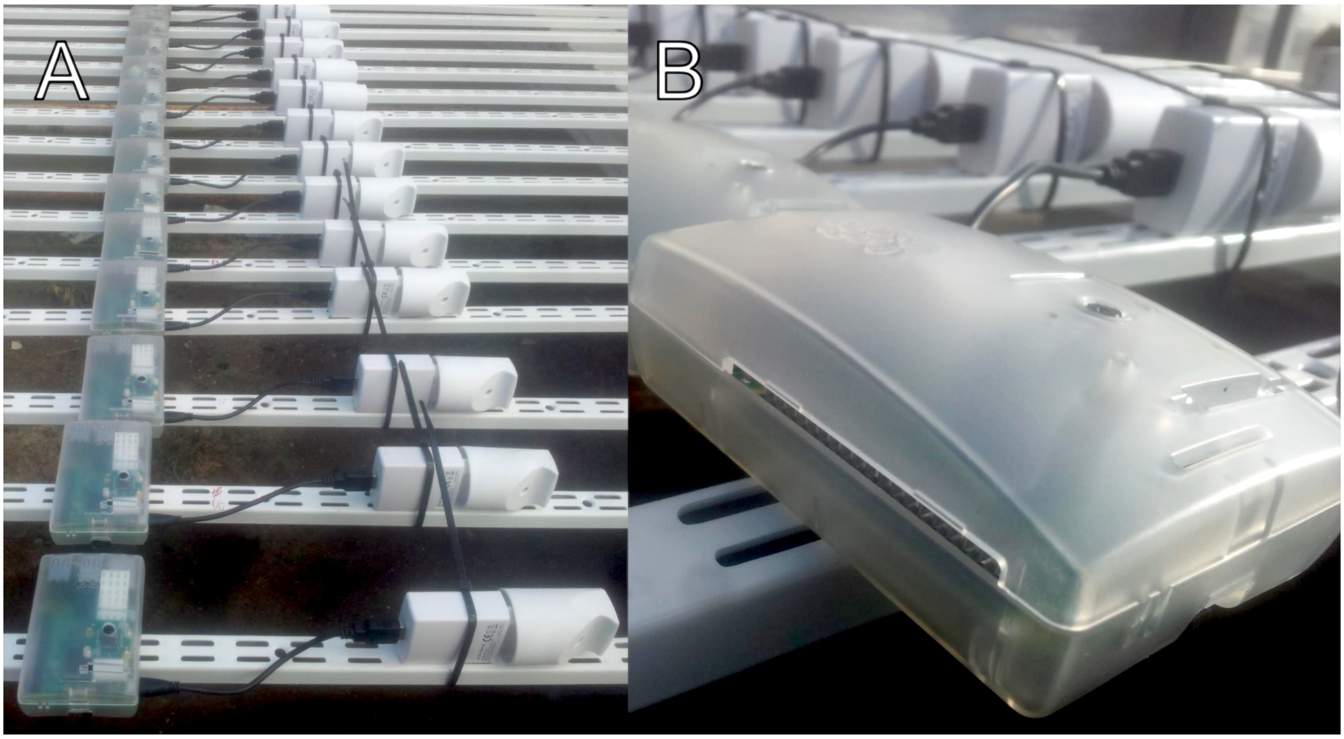

Figure S1. Raspberry Pi camera modules assembly. **(A)** Camera modules mounted to metal U-profiles for easier mounting to rails above the plants. Power Supply with higher technical specifications than needed for operation. USB cable (USB-A to USB Micro-B) is short to reduce electrical resistance and voltage drop. **(B)** Close up of a camera module with a raspberry pi in plastic enclosure. (camera lens visible)

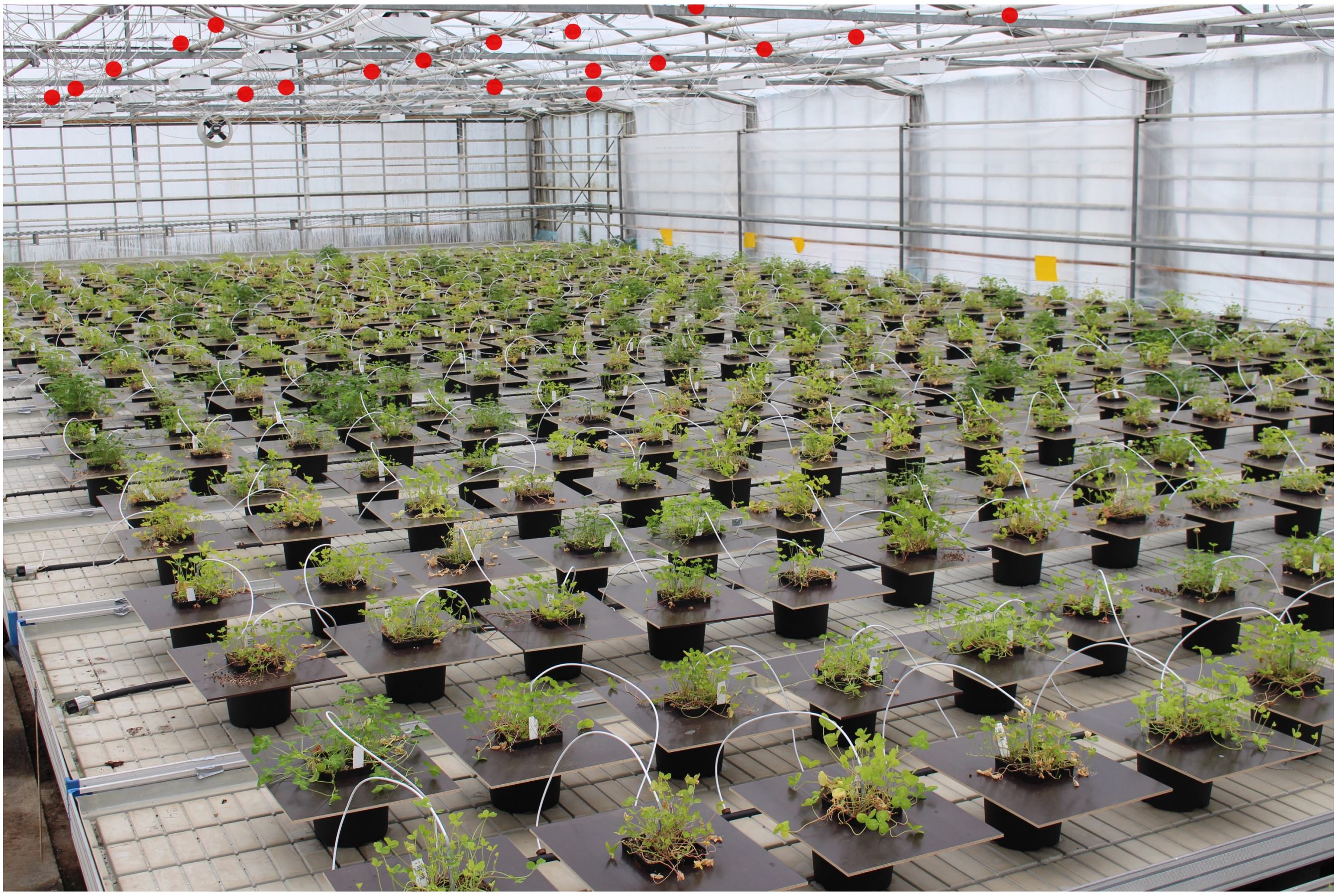

Figure S2. Picture of one side of the greenhouse experiment showing 10 tables. The small camera modules visible on top of the image have been marked with red circles.

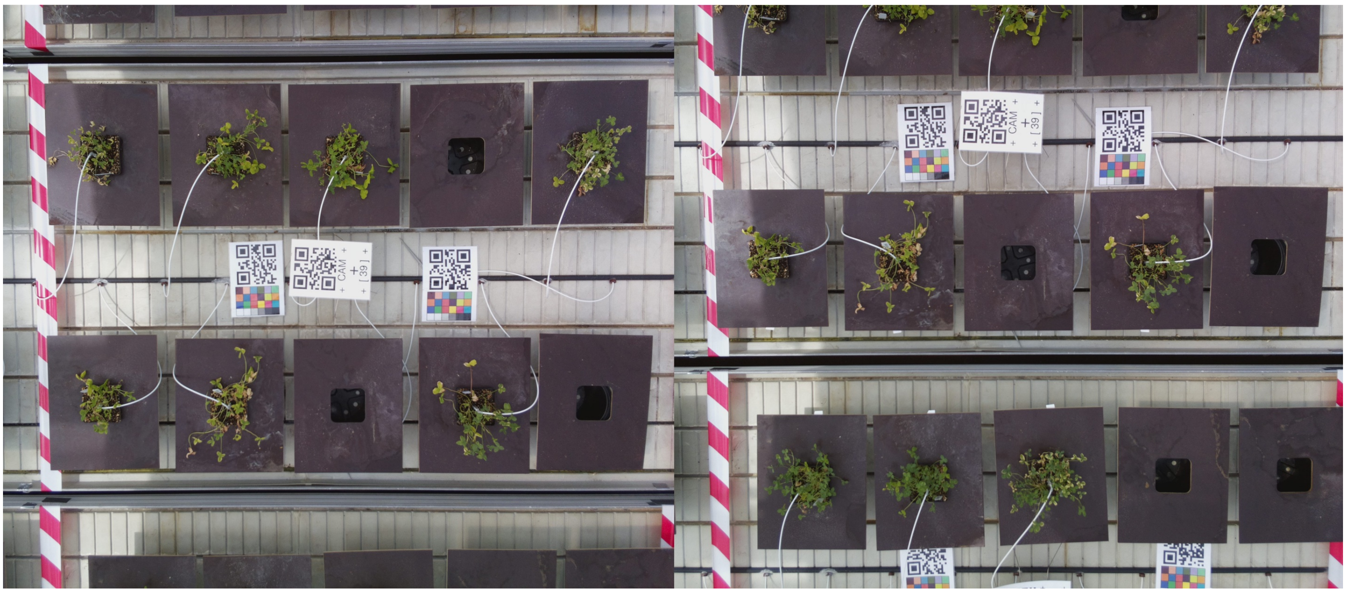

Figure S3. Images 30 minutes apart. Showing the mobility of the tables.

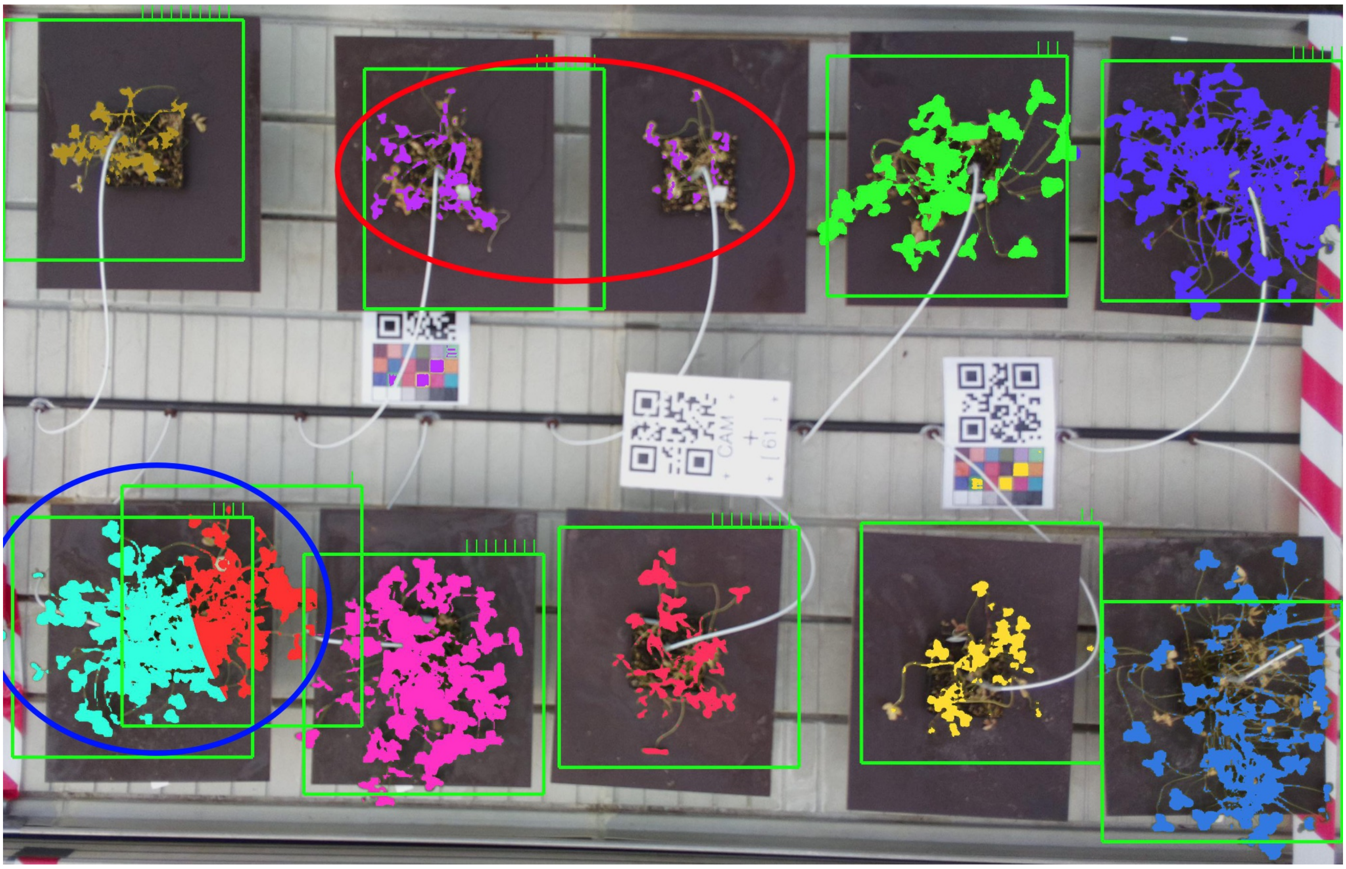

Figure S4. Example when using a clustering solution in the greenhouse setting. This example is using a gaussian mixture to do clustering, where it attempts to divide it into 10 clusters. Indicated by the red circle the clustering joins two plants due to the same size of both, and indicated by the blue circle it splits a plant into two. The green boxes indicate the crop that would be made and the resulting area wherein measurements of the plant would take place.

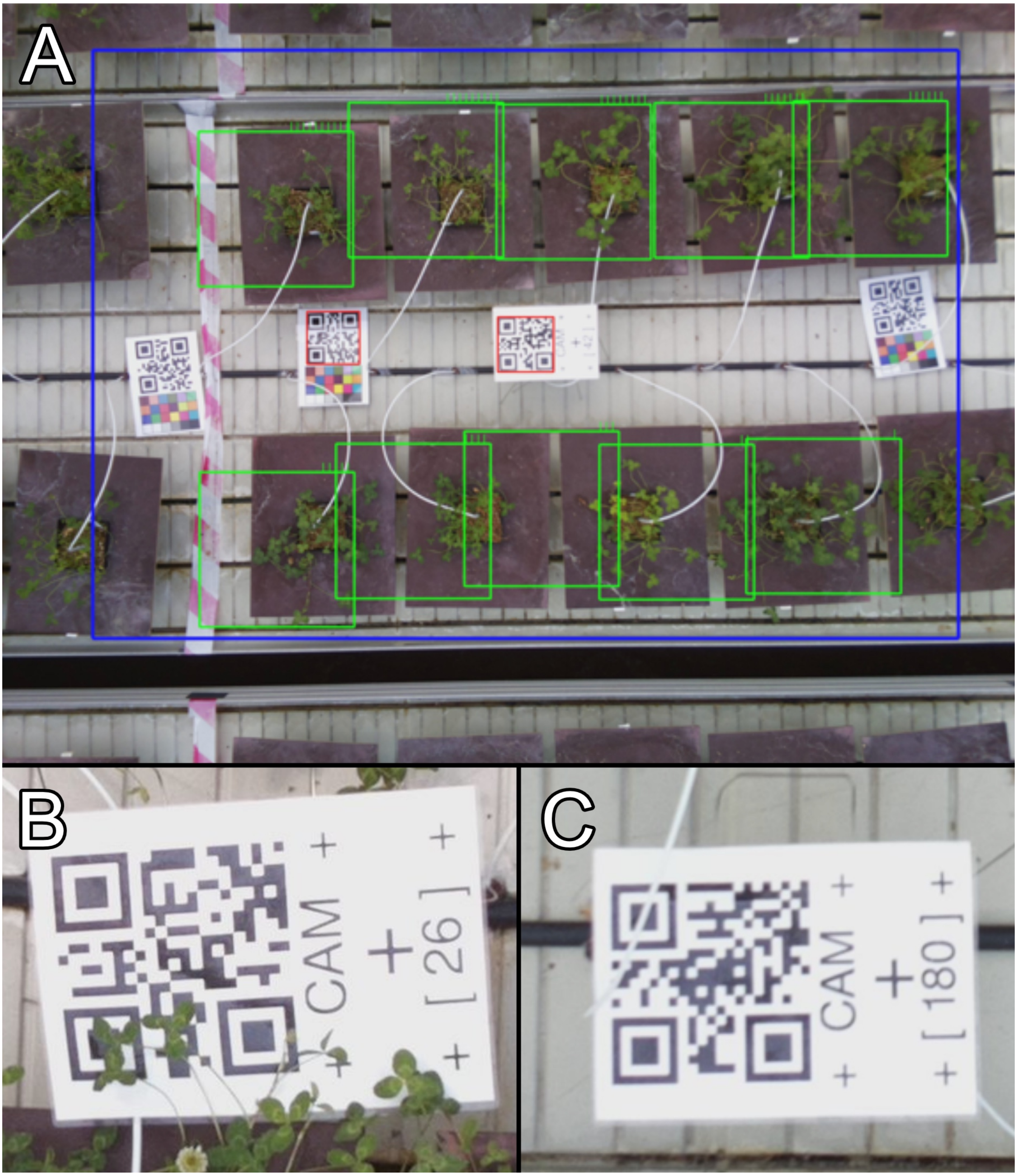

Figure S5. **(A)** Clustering method using markers based on the relative position of the QRcode. Fails to correctly capture the plants due to the QRcode being off center. **(B)** Hard to detect QR code due to overgrowth from the plants blocking the QRcode. **(C)** Hard to detect QR code due to a water tube blocking one of the positioning squares.

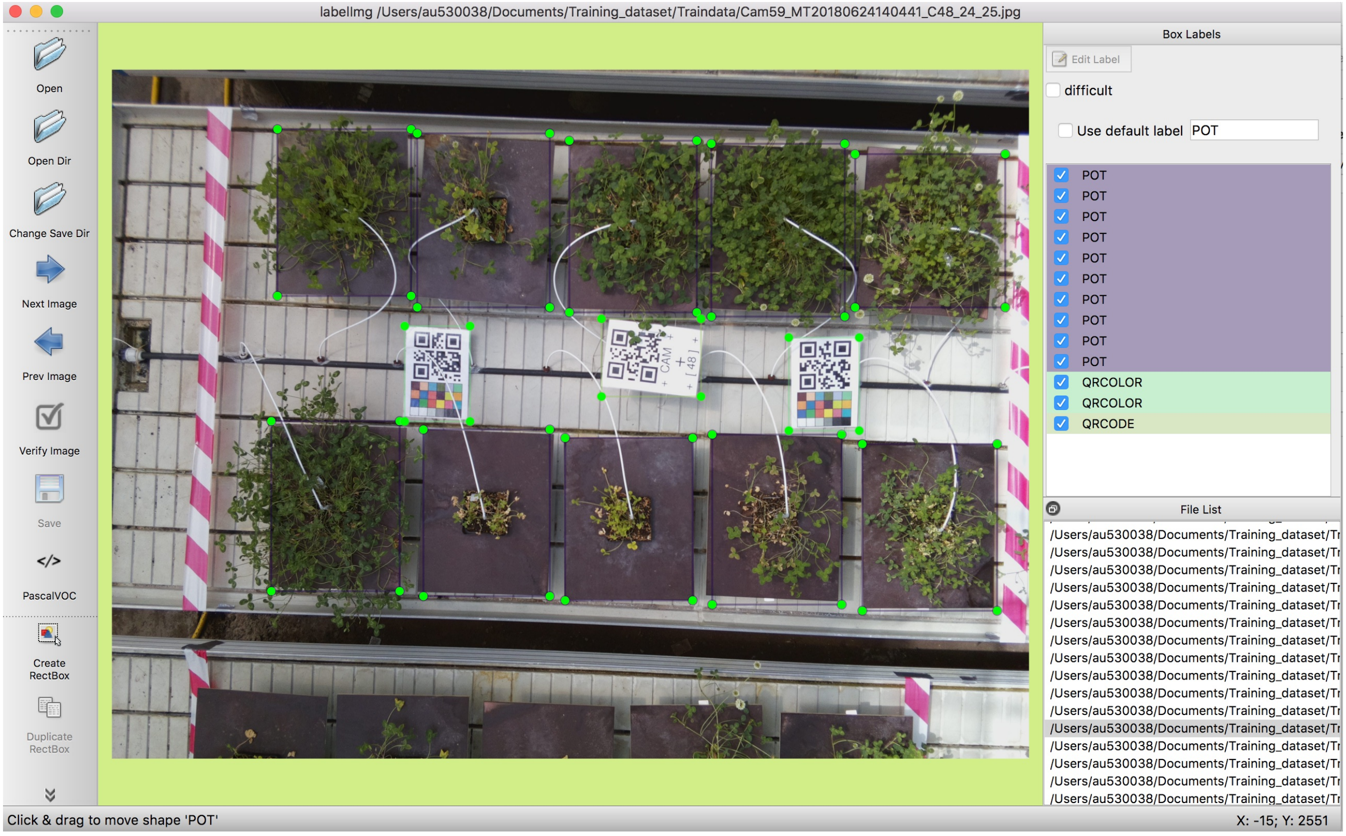

Figure S6. Screenshot from the LabelImg tool. On the right the classifications can be seen with 10 labels named POT corresponding to the 10 plants of interest, 2 named QRCOLOR with the QRcodes with ColorChecker charts, and finally 1 named QRCODE corresponding to the center QRcode used to identify the group.

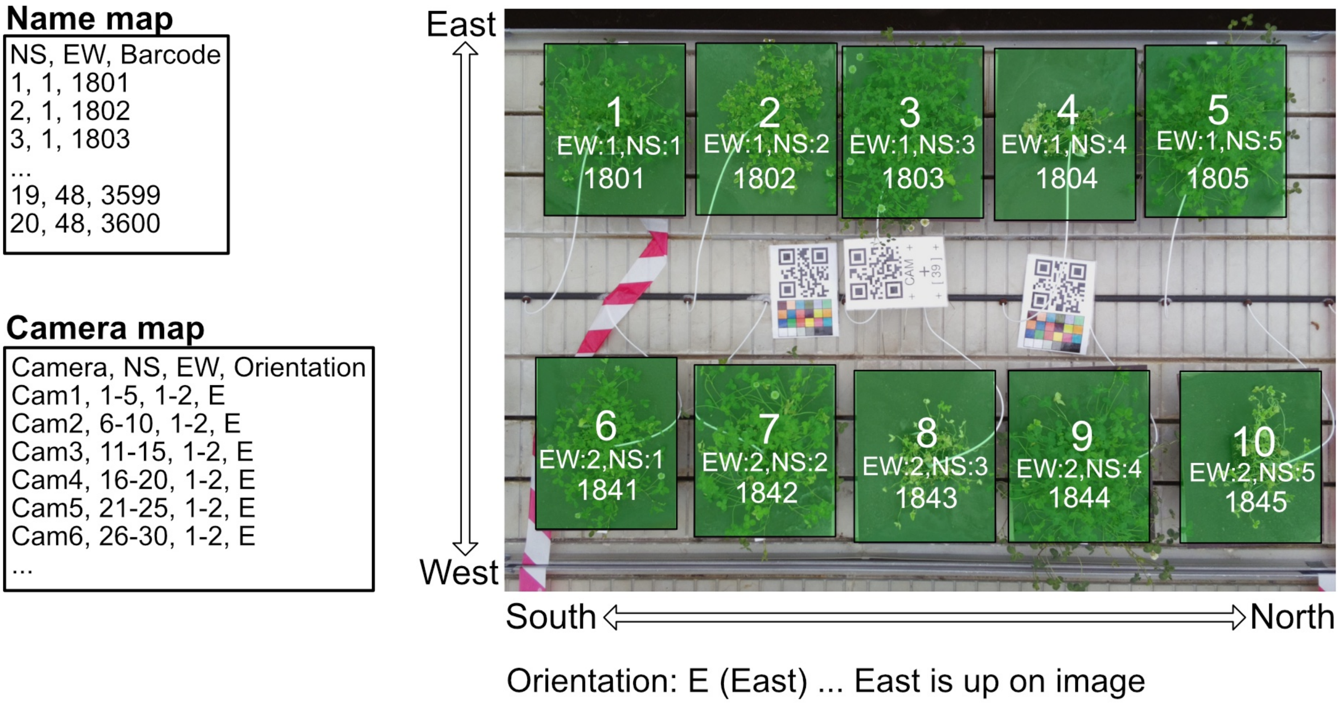

Figure S7. Demonstration of the Pot identification. Name map and Camera map formatted in the CSV (Comma Separated Values) format. Name map contains NS and EW coordinates for each individual pot encoded as a Barcode. Camera map contains the Cam ID and the range of NS and EW coordinates covered by the camera, and in which orientation the camera is placed in the NS and EW space. The example image is represented as Camera 1 (physical camera ID) known from the image filename. The orientation is E (East) since east is up on the image. The numbers on each green block represent the order in which the coordinates are given. The EW:n,NS:n describe the given coordinate to the pot and number below is the corresponding barcode given to the pot.

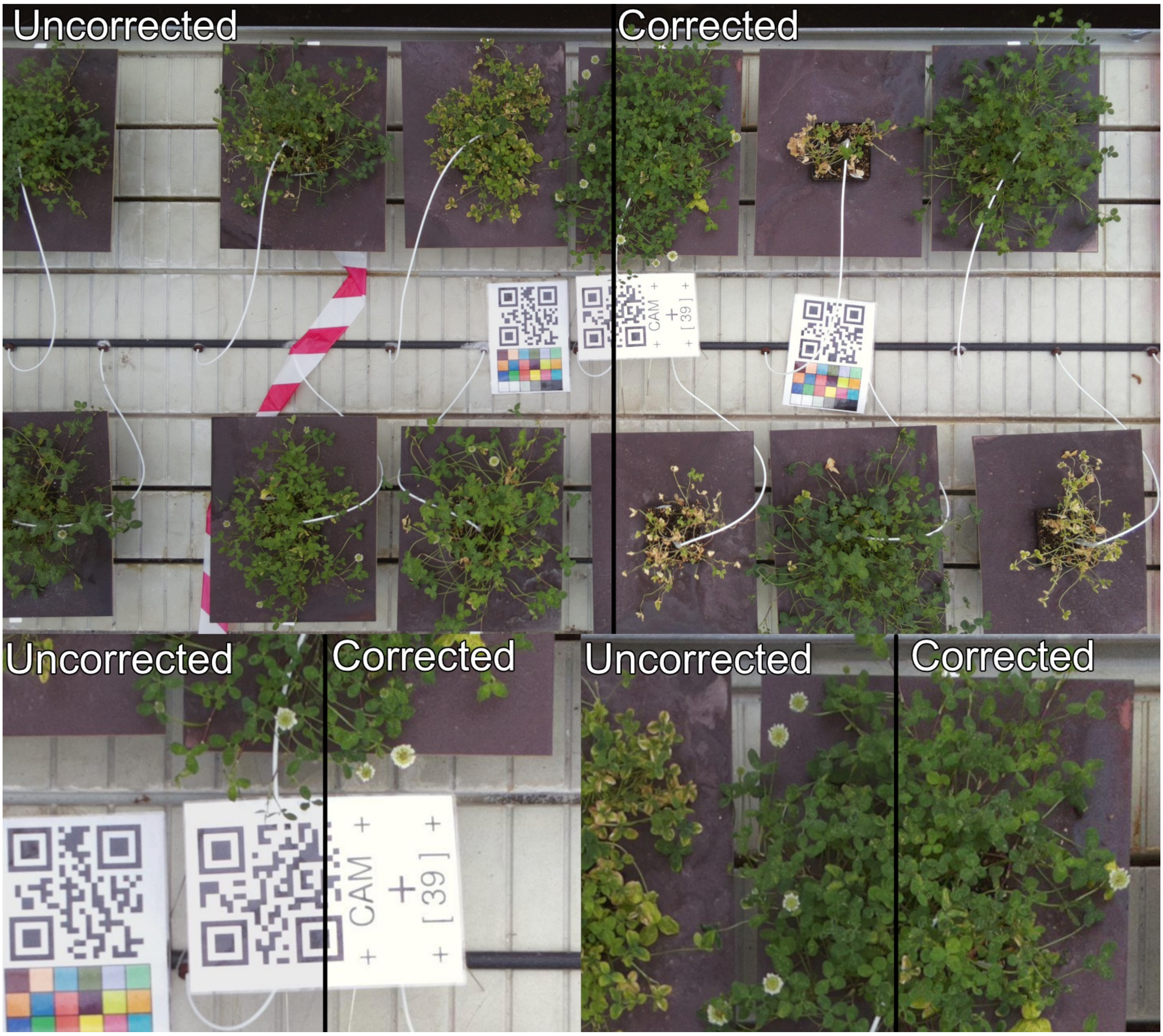

Figure S8. Color correction demonstration using white balance correction based on the QR code white color. Left side is uncorrected having a darker tint and the right side is corrected into being slightly brighter. Below are zoomed in parts of the QR code and the plant above it.

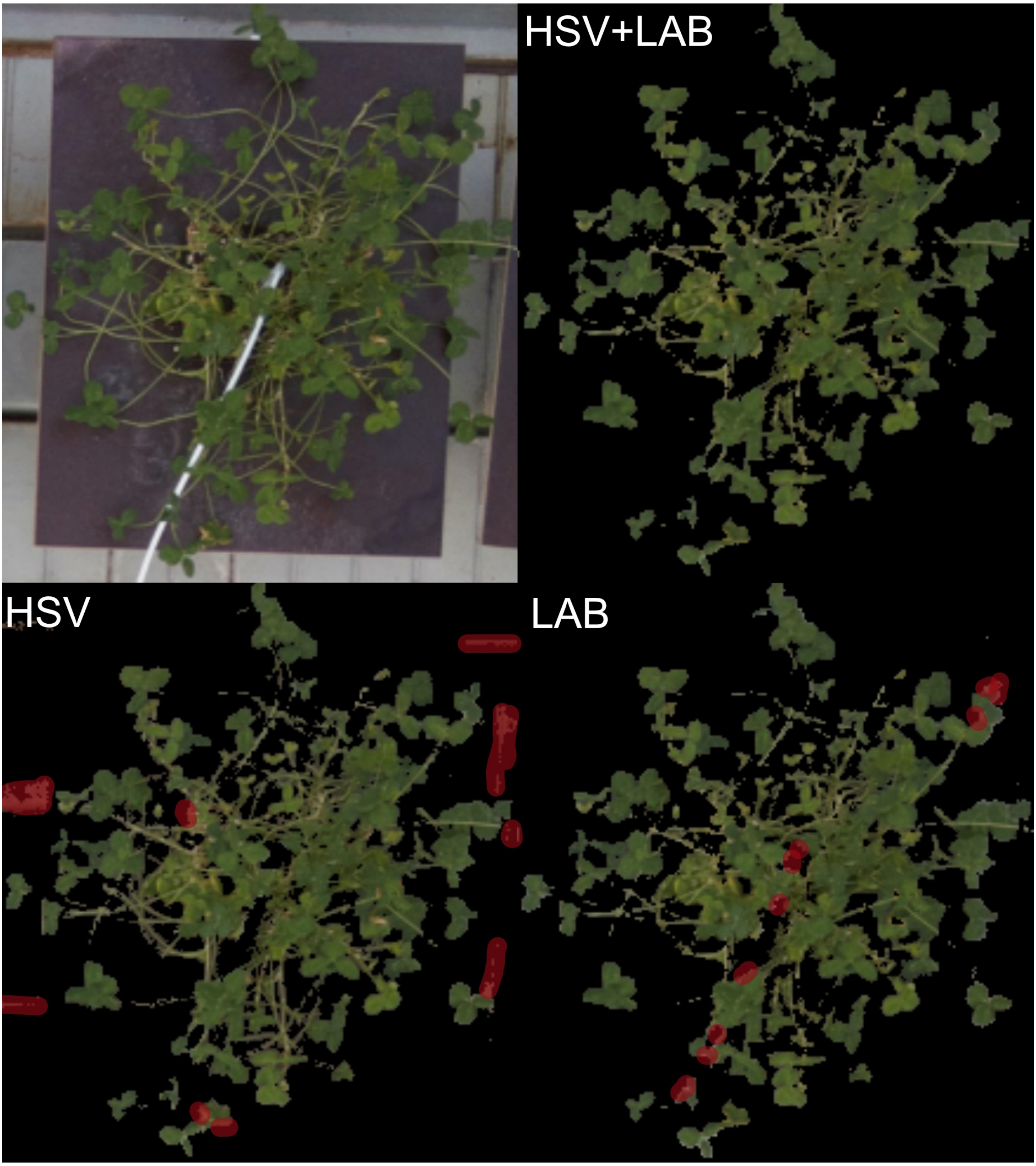

Figure S9. Demonstration of the plant area detection. Top left panel shows the unprocessed input image, top right panel shows the combined HSV and LAB threshold. Bottom left panel shows the HSV threshold result, and the bottom right panel shows the CIELAB threshold. The red regions show areas the HSV and LAB thresholds detect wrong pixels, which are removed when combining the thresholds.

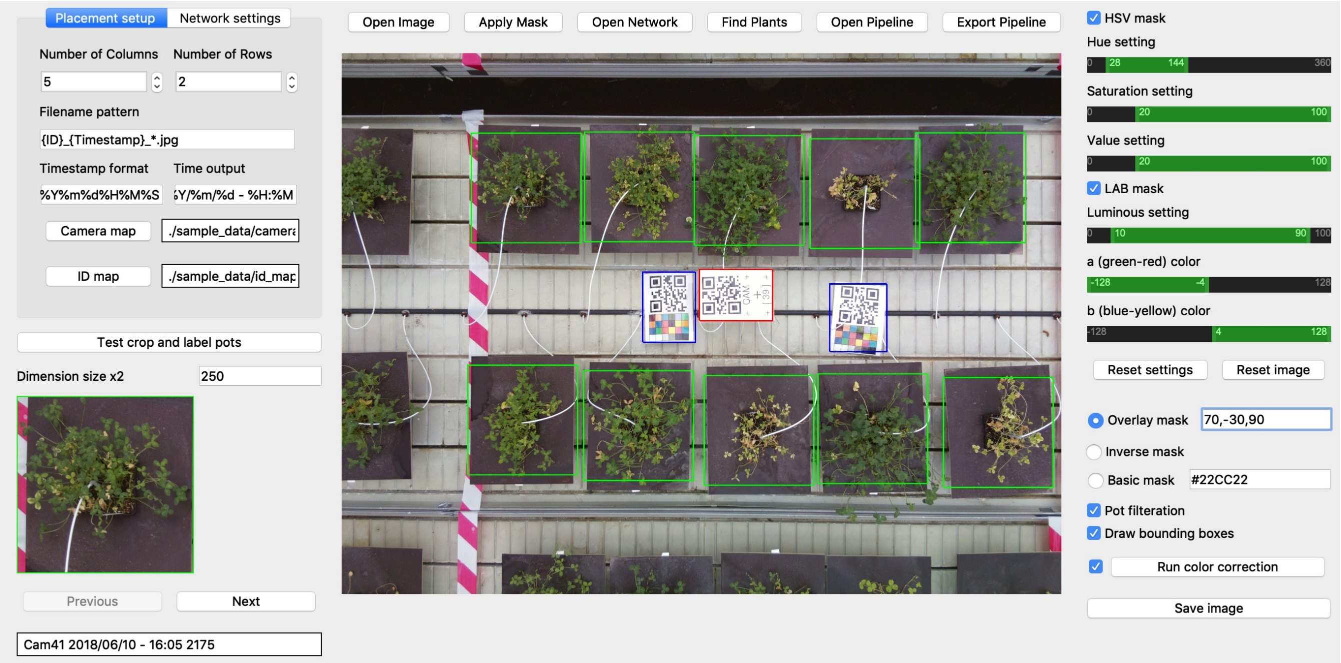

Figure S10. Screenshot of the pipeline planner GUI. The Main toolbar is positioned over the image, allowing the opening of images, applying the mask, and testing the network (Find Plants). The right panel contains visual settings for changing the mask settings and drawing options. Left panel contains options for the placement setup and organisation. The bottom left area contains options for testing the pipeline on the present image.

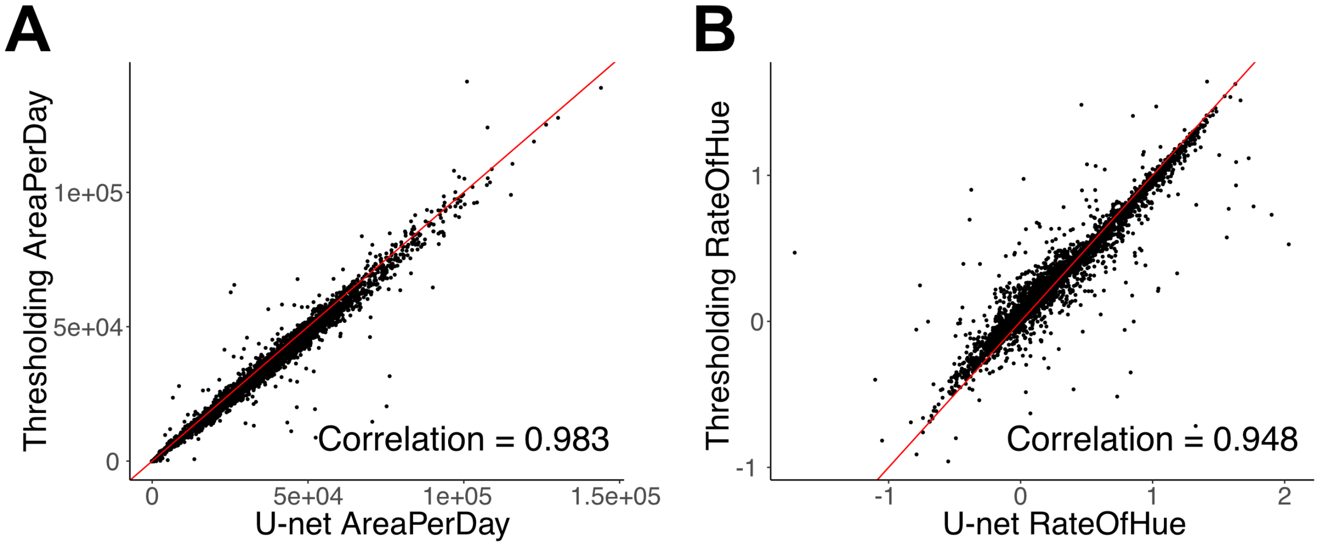

Figure S11. **(A)** U-net and thresholding correlation of the AreaPerDay measurement estimated from the growth curves. **(B)** U-net and thresholding correlation of the RateOfHue estimated from the greenness measurements from the image data.

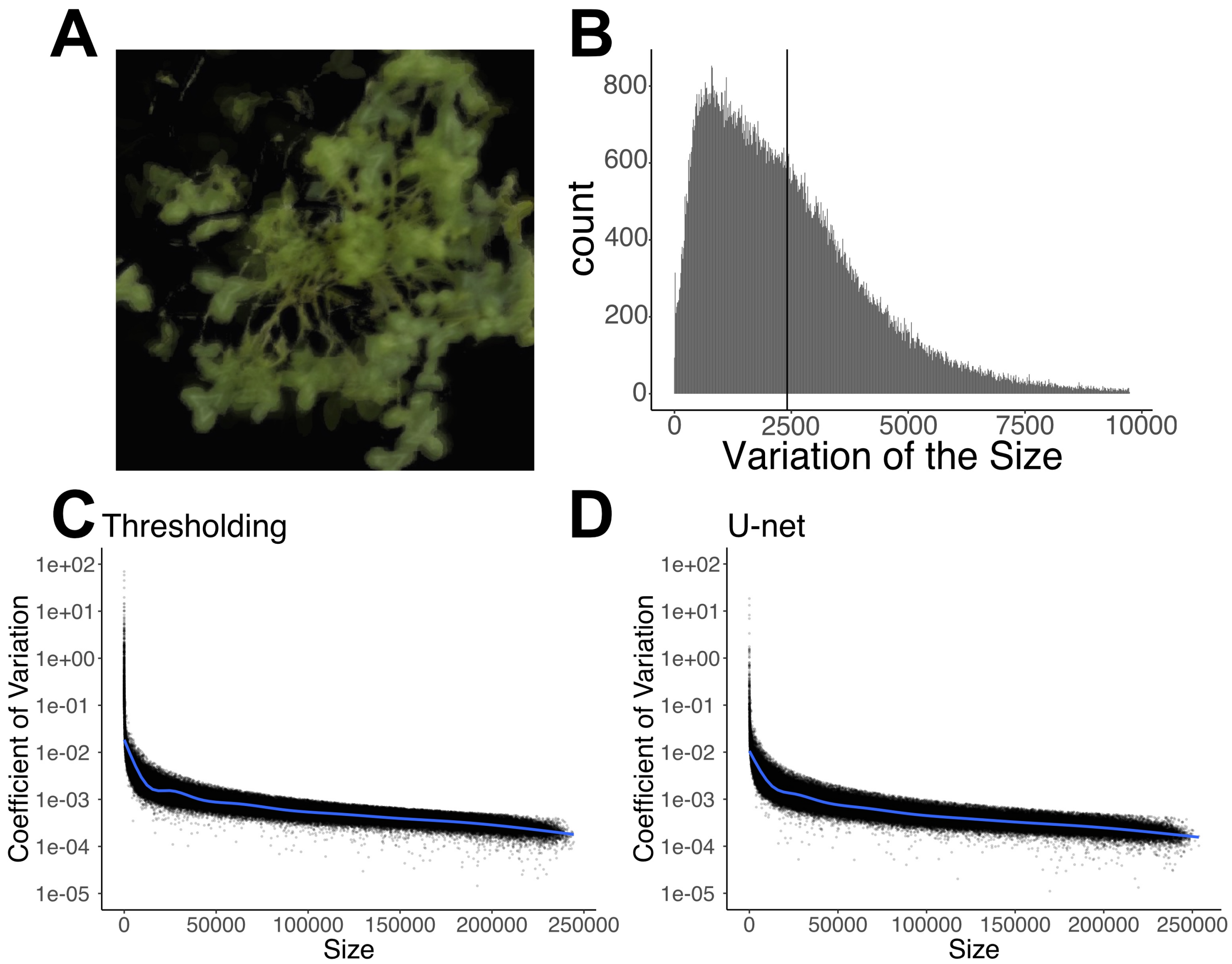

Figure S12. **(A)** Transparent overlay of masked images from a single day. The images shows that the variation within a day is largely due to the movement of the plants throughout the day. **(B)** Histogram of all the measurements showing the variation in the size. The majority have low variation, while a few have very high variation likely due to problems with light or improper detection. **(C & D)** Coefficient of variation plotted against plant area size in pixels, with segmentations made using thresholding **(C)** and U-net **(D)**.

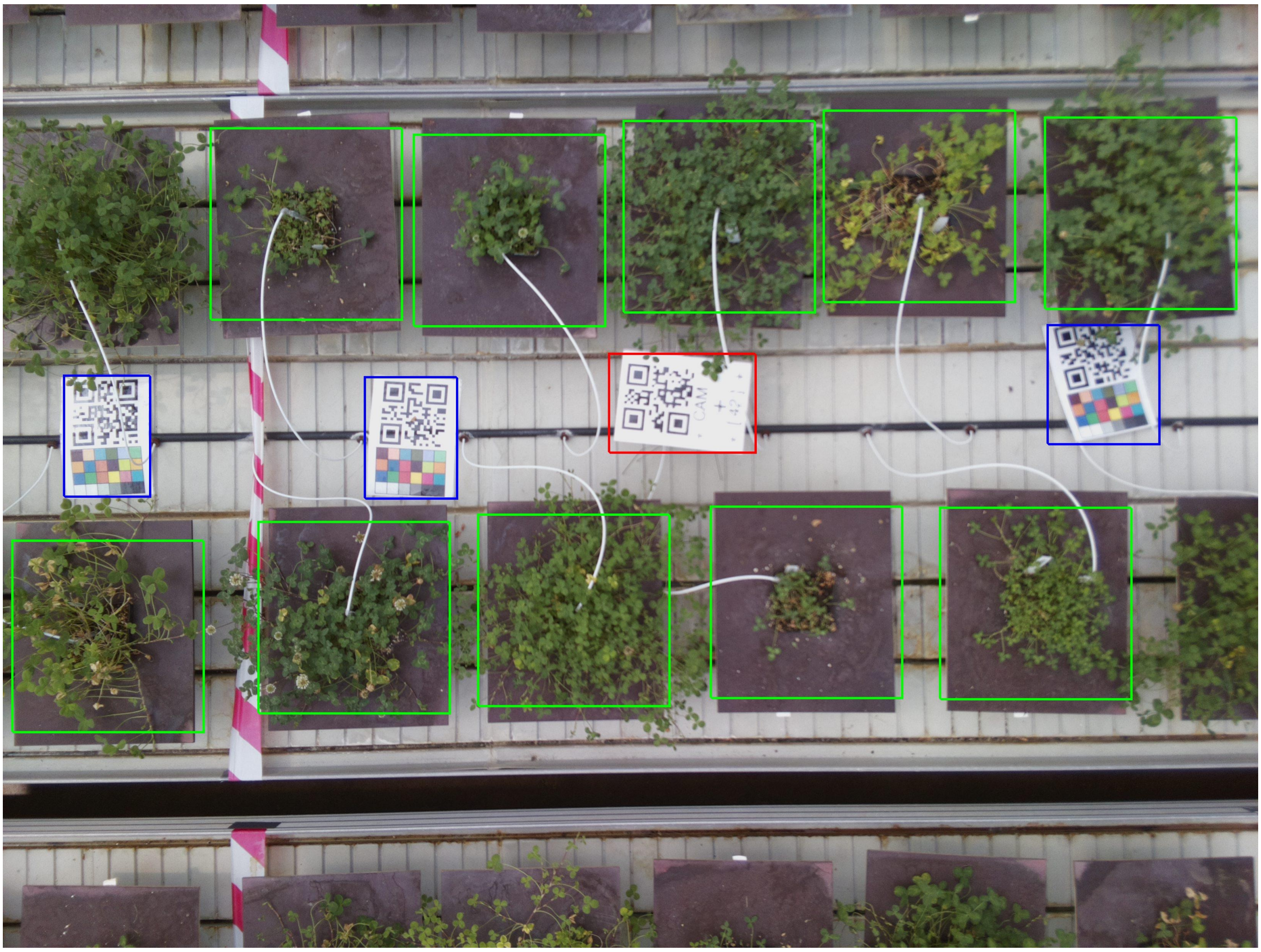

Figure S13. The intended plants were not placed correctly, bottom right is almost completely out of frame. This causes the entire bottom row to be wrongly identified.

2.2 Tables

Table S1. The distribution of the uptimes before and after the backup has been introduced. The uptimes have been grouped into 5 classes of 5% starting from 75% up to 100%. The number of cameras is the count of cameras within the group of uptimes.

	**Without Backup**				
Uptimes	75-80 %	80-85 %	85-90 %	90-95 %	95-100 %
Number of cameras	8	11	161	0	0
	**With Backup**				
Uptimes	75-80 %	80-85 %	85-90 %	90-95 %	95-100 %
Number of cameras	0	0	3	9	168
